# Persistent motor dysfunction despite homeostatic rescue of cerebellar morphogenesis in the *Car8 waddles* mutant mouse

**DOI:** 10.1186/s13064-019-0130-4

**Published:** 2019-03-12

**Authors:** Lauren N. Miterko, Joshua J. White, Tao Lin, Amanda M. Brown, Kevin J. O’Donovan, Roy V. Sillitoe

**Affiliations:** 1 Department of Pathology and Immunology, Dan Duncan Neurological Research Institute of Texas Children’s Hospital, 1250 Moursund Street, Suite 1325, Houston, TX 77030 USA; 2 Department of Neuroscience, Dan Duncan Neurological Research Institute of Texas Children’s Hospital, 1250 Moursund Street, Suite 1325, Houston, TX 77030 USA; 3 Program in Developmental Biology, Baylor College of Medicine, Dan Duncan Neurological Research Institute of Texas Children’s Hospital, 1250 Moursund Street, Suite 1325, Houston, TX 77030 USA; 4 Jan and Dan Duncan Neurological Research Institute of Texas Children’s Hospital, 1250 Moursund Street, Suite 1325, Houston, TX 77030 USA; 5 Department of Chemistry and Life Science, United States Military Academy, West Point, New York, 10996 USA; 6 Burke Neurological Institute, Weill Cornell Medicine, White Plains, 10605 USA

**Keywords:** Purkinje cell, Granule cell, Proliferation, Stem cells, Lobule, Ataxia, Tremor, Dystonia

## Abstract

**Background:**

Purkinje cells play a central role in establishing the cerebellar circuit. Accordingly, disrupting Purkinje cell development impairs cerebellar morphogenesis and motor function. In the *Car8*^*wdl*^ mouse model of hereditary ataxia, severe motor deficits arise despite the cerebellum overcoming initial defects in size and morphology.

**Methods:**

To resolve how this compensation occurs, we asked how the loss of carbonic anhydrase 8 (CAR8), a regulator of IP3R1 Ca^2+^ signaling in Purkinje cells, alters cerebellar development in *Car8*^*wdl*^ mice. Using a combination of histological, physiological, and behavioral analyses, we determined the extent to which the loss of CAR8 affects cerebellar anatomy, neuronal firing, and motor coordination during development.

**Results:**

Our results reveal that granule cell proliferation is reduced in early postnatal mutants, although by the third postnatal week there is enhanced and prolonged proliferation, plus an upregulation of Sox2 expression in the inner EGL. Modified circuit patterning of Purkinje cells and Bergmann glia accompany these granule cell adjustments. We also find that although anatomy eventually normalizes, the abnormal activity of neurons and muscles persists.

**Conclusions:**

Our data show that losing CAR8 only transiently restricts cerebellar growth, but permanently damages its function. These data support two current hypotheses about cerebellar development and disease: (1) Sox2 expression may be upregulated at sites of injury and contribute to the rescue of cerebellar structure and (2) transient delays to developmental processes may precede permanent motor dysfunction. Furthermore, we characterize *waddles* mutant mouse morphology and behavior during development and propose a Sox2-positive, cell-mediated role for rescue in a mouse model of human motor diseases.

**Electronic supplementary material:**

The online version of this article (10.1186/s13064-019-0130-4) contains supplementary material, which is available to authorized users.

## Introduction

The cerebellum controls motor coordination, motor learning, posture, and balance. Therefore, damage to its circuits causes a number of motor disorders, such as ataxia, dystonia, and tremor [1–7]. A range of Purkinje cell alterations may be at the center of all cerebellar disease phenotypes, irrespective of whether the causative insult occurs during development or in adulthood [8]. In adults, Purkinje cells typically contribute to movement defects because they degenerate, whereas during development they impact motor function by failing to properly assemble the circuitry [5, 9–13]. This study tests how motor dysfunction arises even when the assembly of cerebellar structure is rescued, and no obvious degeneration occurs.

The functional architecture of the cerebellum is established through multiple morphogenetic, patterning, and remodeling processes [14]. These processes include lobule formation, zonal patterning of zebra-like striped compartments, and afferent fiber synaptogenesis. Each of these processes operates within a very dynamic environment, whereby the cerebellum rapidly grows in size and the folds deepen to form sub-divisions that generate the recognizable foliation pattern in adults [15]. Cerebellar growth and foliation are dependent on several molecular cues including sonic hedgehog, engrailed1/2, and Fgf signaling [16–18]. Regardless of the initial genetic defect, granule cell proliferation is almost always affected if cerebellar size and growth are impacted. And because of bidirectional signaling between developing granule cells and Purkinje cells [19], when one cell type is affected the other is as well. Disrupting Purkinje cell morphogenesis can also cause dendrite anomalies [20] and miswiring of afferents [21–23], which both lead to circuit firing problems [24, 25]. From an experimental perspective, it has been challenging to parse out how developmental defects impact the onset of motor dysfunction in animal models of movement disease. Major reasons for this challenge are that the affected Purkinje cells can degenerate, undergo cell death, or even look normal but function poorly due to a compromised circuit. Such variations call into question how developmental mechanisms intersect with structure, functional circuitry, and behavior in ataxia and related disorders [26–29].

*Car8*^*wdl*^ mice are ideal for testing how morphogenesis and wiring impact motor dysfunction [30]. In the brain, CAR8 protein is expressed predominantly in Purkinje cells. Its expression is initiated during embryogenesis and maintained into adulthood [31, 32]. CAR8 belongs to a family of zinc metalloenzymes that catalyze the reversible hydration of CO_2_ [33], although CAR8 lacks the catalytic domain that would make it an active carbonic anhydrase [31]. It does, however, bind to inositol 1,4,5-triphosphate receptor type 1 (IP3R1), where it has the proposed effect of decreasing the affinity of IP3 for its receptor [34]. *Car8*^*wdl*^ mutant mice have ataxia, tremor, and appendicular dystonia, with cerebellar microcircuit abnormalities [30, 35] supposedly occurring without gross anatomical defects [36]. In humans, mutations in the orthologous gene, *CA8*, also cause ataxia and a predisposition for quadrupedal locomotion [37, 38]. To better understand how the motor deficits arise and then persist without any accompanying severe morphological defects, we used a combination of genetics, gene expression analysis, in vivo electrophysiology, and mouse behavior to follow cerebellar morphogenesis and function in postnatal developing *Car8*^*wdl*^ mice. We uncover transient defects in cerebellar size, Purkinje cell morphology, and granule cell proliferation during development in *Car8*^*wdl*^ mice. Although most of the structural deficits are corrected by weaning, neural circuit function remains impaired and behavior deficits persist in adulthood.

## Methods

### Animals

*Car8*^*wdl*^ mutant mice (Stock 004625), C57BLKS/J control background strain, and *NpyGFP* mice (B6.Cg-Tg(Npy-MAPT/Sapphire)1Rck/J, Stock 008321) were purchased from The Jackson Laboratory (Bar Harbor, ME) and then maintained in our animal colony at Baylor College of Medicine. We bred the control and mutant mice using timed pregnancies, and we designated noon on the day a vaginal plug was detected as embryonic day (E) 0.5 and the day of birth as postnatal day (P) 0. We used a standard PCR genotyping protocol to differentiate the mutants from the controls using the same primer sequences as previously described [30, 36]. Mice of both sexes were studied. They had food and water ad libitum. All animal studies were carried out under an approved IACUC animal protocol according to the institutional guidelines at BCM.

### Perfusion, basic histology, and tissue staining procedures

After being anesthetized under 2,2,2-tribromoethanol (Avertin), mice (ages P5, P10, P15, P17, P20, P180, and P360 adults) were transcardially perfused, first with 0.1 M PBS (pH 7.2) then with 4% paraformaldehyde (PFA). Dissected brains from the perfused mice were post-fixed in 4% PFA for at least 24 h then transferred onto 2% agar for whole mount imaging, transferred sequentially through a series of sucrose solutions (18 and 30%) for cryoprotection, or embedded in paraffin. Whole mount images were taken using Zeiss AxioZoom VI6 to compare cerebellar size and lobulation pattern across 5 ages (P5, P10, P15, P20, Adult) in *Car8*^*wdl*^ and C57BLKS/J mice. Cryoprotected and frozen tissues were cut sagittally on a cryostat into 40 μm (or 80 μm, as for Golgi-Cox staining) sections and stored at 4 °C, free-floating in PBS. Midline sections of the cerebellum were either stained following Golgi-Cox and immunohistochemistry staining protocols published previously [39–42] or following a modified protocol for manual hematoxylin and eosin (H&E) staining of frozen tissue [43]. Tissues embedded in paraffin first were dehydrated by overnight incubations in 70% ethanol, 95% ethanol, 100% ethanol, and chloroform. Dehydrated tissue was then transferred to plastic cassettes, where they soaked in 2 changes of paraffin (Paraffin #1 for ~ 18 h, Paraffin #2 for ~ 2 h) in a 65 °C oven. Hot paraffin covered the brains and using forceps, brains were positioned on their sides for sagittal sectioning. Tissue was left overnight at room temperature until the paraffin solidified. Paraffin-embedded tissues were cut on a microtome into 10 μm sections, mounted onto slides from a warm water bath, and transferred to a 65 °C oven overnight for ~ 16–18 h.

We performed the Golgi-Cox staining [44] with a modified procedure using the FD Rapid GolgiStain Kit (FD Neurotechnologies, Inc) on 80 μm-thick tissue sections that were processed as described by the manufacturer using a free-floating approach. Stained tissue sections were imaged using a Zeiss AxioImage.M2 microscope (additional details below).

Tissues stained via immunohistochemistry were first incubated in 10% Normal Donkey Serum (NDS, Sigma) or 10% Normal Goat Serum (NGS) blocking solution (0.1% Tween 20 and 0.1 M PBS) for 1–2 h then were incubated with primary antibodies in new aliquots of 10% NDS or 10% NGS blocking solution overnight (~ 16–18 h). A Calbindin D-28 K monoclonal mouse antibody (Swant #300) was used to visualize Purkinje cell soma, axons, and dendrites at a 1:10,000 dilution. A CAVIII polyclonal rabbit antibody (Santa Cruz sc67330) differentiated Purkinje cells with carbonic anhydrase 8 (CAR8) from other cells that lacked this metalloenzyme at a 1:500 dilution. To visualize proliferating cells, a polyclonal mouse Ki67 antibody (BD Pharmingen #55069 Lot 33,112,568) was used at a 1:400 dilution with NeuroTrace 435/455 Blue Fluorescent Nissl Stain (ThermoFisher Scientific #N21479) or DAPI (Vectashield Antifade Mounting Medium with DAPI #H-1200) to distinguish the External Granular Layer (EGL). To visualize mitotically active cells, a polyclonal mouse PH3 (Ser10) antibody (Cell Signaling 9701S Lot 16) was used at a 1:200 dilution with NeuroTrace 435/455 Blue Fluorescent Nissl Stain (ThermoFisher Scientific #N21479) or DAPI (Vectashield Antifade Mounting Medium with DAPI #H-1200). Sox2 (Invitrogen PA1–094 Lot RH235771 and Millipore Sigma Cat. No. AB5603) polyclonal antibodies were used at a 1:50 and 1:200 dilution, respectively, to determine if cerebellar stem cells (or granule cell precursors expressing Sox2) were present in the P5 and P10 EGL. There were no obvious differences in Sox2 staining between the two antibodies on the cerebellar tissue tested. To evaluate the zonal patterning of Bergmann glia and Purkinje cells during *Car8*^*wdl*^ development, a rabbit polyclonal anti-HSP25 (StressGen #SPA-801) and a chicken anti-GFP (Abcam Cat. No. ab13970) were used at a 1:500 and a 1:2000 dilution, respectively. Tissue was rinsed 3–4 times for 5 min each with 0.1 M PBS before incubating the tissue with fluorescent Alexa 488-, 555-, or 647- immunoglobins (Invitrogen Molecular Probes Inc., Eugene, OR, USA #A-21202, #A-31572, and #A-31573) diluted 1:1500 in new aliquots of 10% NDS or 10% NGS blocking solution for 2 h. Following additional 4 X 5-min rinses, the tissues were mounted onto electrostatically coated glass slides, coverslipped with FLUORO-GEL (with Tris Buffer) and then imaged using a Zeiss AxioImage.M2 microscope (additional details below).

Tissues stained with H&E followed one of two procedures. If tissues were sectioned on the cryostat, they were first mounted onto slides and allowed to dry for approximately 5 min. Once there appeared to be no more moisture on the adhered tissues, the slides were placed in hematoxylin for approximately 5 min, transferred to lithium until the sections turned a deep blue, then placed in eosin for about 1 min. Sections were next subjected to a series of ethanol washes for dehydration (70, 95, 100, 100, 100%, xylene, xylene). Tissues were dipped 20 times in both 70 and 95% ethanol, 10 times in the first change of 100% ethanol, then 5 times in the last 2 changes of 100% ethanol. Slides were transferred to the first change of xylene for 3–5 min, and then moved to the second change of xylene for 3–5 min. Slides were then cover-slipped with Cytoseal and imaged using a Zeiss AxioZoom VI6 microscope after at least 24 h in the fume hood. Paraffin-embedded tissues were left on slides overnight in a 65 °C oven. To remove the paraffin, slides containing the sections were relayed from 2 changes of xylene, to 2 changes of 100% ethanol, to 95% ethanol, and lastly to 85% ethanol. In each of the solutions, slides were immersed for 2 min. After the 85% ethanol wash, slides were placed in hematoxylin for approximately 3 min, transferred to lithium until the sections turned a deep blue, then placed in eosin for about 30 s. Like the cryostat-sectioned tissues, after eosin staining, paraffin-sectioned tissues were processed through the same dehydration series as indicated above. Paraffin-embedded tissue sections were also cover-slipped with Cytoseal and imaged using a Zeiss AxioZoom VI6 microscope approximately 24 h after staining.

### In vivo EdU labeling

The protocol followed was adapted from Mead and Lefebvre (2014) [45]. In vivo EdU labeling was performed using the Click-iT EdU 488 Imaging Kit (Invitrogen Catalog #C10337). P5 and P10 mutant and control pups were injected either subcutaneously (P5) or intraperitoneally (P10) with 100 μL of 5-ethynyl-2′-deoxyuridine (EdU) per 10 g. After 4 h, the injected pups were anesthetized with isoflurane, and then decapitated to remove the brain. The brain was immersed in 4% paraformaldehyde (PFA) for 48 h at 4 °C and then transferred to a demineralization solution consisting of 1% PFA and 0.5 M Tris-EDTA (pH 8) at 4 °C. The demineralization solution was replaced with fresh aliquots every other day for 7 days. After 7 days, the brains were cryoprotected, embedded, and sectioned following the methods described above (see *Perfusion, basic histology, and tissue staining procedures*). At least 3 sections from 4–5 mutants and 5–6 controls at P5 and P10 were mounted onto electrostatically coated glass slides and dried on the bench for 1 h at room temperature (22–25 °C). The mounted tissue was washed 3 times in 1X PBS for 2 min each before being incubated in Click-iT reaction cocktail for 30 min. The Click-iT reaction cocktail consisted of a 1X Click-iT reaction buffer, copper sulfate (CuSO_4_), Alexa Fluor 488 azide, and 1X reaction buffer additive. Following incubation, the tissue was washed twice for 5 min each in 1X PBS. Slides were coverslipped with FLUORO-GEL (with Tris Buffer) and then imaged using a Zeiss AxioImage.M2 microscope (additional details below).

### Image acquisition and quantification

Photomicrographs of some of the *NpyGFP* reporter stained tissue sections were captured using a Leica DFC 360 FX camera mounted on a Leica DM5500 microscope. Images of the rest of the tissue sections were acquired and analyzed using Leica Application Suite software. For the H&E and fluorescently stained tissue sections, at least 2 sections from at least 3 animals were analyzed per developmental age studied (P5, P10, P15, and P20). Sagittal tissue sections containing all 10 lobules were selected within ±0.36 mm from the midline [46], as determined by the lack of cerebellar nuclei seen in the white matter. The medial-lateral coordinates of the tissue sections selected were matched across genotype for each of the observed ages. The H&E stained tissues that were used to quantify cerebellar size were imaged on the Zeiss AxioZoom VI6. The areas of the sagittal cross-sections were calculated using ImageJ software. The fluorescently stained tissues that were assessed for CAR8, Ki67, calbindin, PH3, Sox2, and EdU expression were imaged on the Zeiss AxioImage.M2 using the Apotome and/or Z-stack features. ImageJ was used for automated and manual counting of Ki67-positive, PH3-positive, EdU-positive cells in the EGL of lobules V and VI. Lobules V and VI were selected for analysis due to their roles in locomotion [47–51]. The long, straight regions of the cerebellar cortex in these lobules (particularly the depths within the primary fissure) are also ideal for anatomical analysis since this architecture allows the acquisition of consistent measurements. To estimate the density of PH3-positive cells in P5 and P10 animals, cells were therefore counted from lobules V and VI. ImageJ was also employed to measure ML thickness, EGL area, Purkinje cell dendritic tree length, width, area, and to manually count the number of primary (i.e. from the soma) and secondary (i.e. from the primary branches) dendrites on Purkinje cells in lobules V and VI. The ML thickness was defined as the distance from the Purkinje cell layer (PCL) to the EGL. Therefore, the distance between the Purkinje cell soma and the concentrated DAPI that demarcates the EGL were measured from 3 locations (left, center, right) per ML per section. The EGL was defined by DAPI staining and its boundaries were delineated from a lack of Calbindin staining and the surface of the lobule. The area of 2 EGLs was measured per tissue section. The inner EGL refers to the ~ 27,000 μm^2^ (P5) or the ~ 14,000 μm^2^ (P10) of EGL immediately adjacent to the ML (i.e. ~ 50% of the EGL as characterized by an absence of calbindin and a sparser labeling of DAPI). Here, the area of inner EGL was calculated by roughly dividing the total control EGL area at P5 and P10 (Fig. 5) by 2. For the Purkinje cell morphology measurements, 15 non-neighboring cells were chosen from different regions of each section, which were taken from 3 different mutants and controls at P5 and P10. At P15, 8 Purkinje cells were chosen from each section belonging to 3 different mutants and controls for quantification. The number of primary and secondary branches were checked and confirmed in ImageJ through adjusting the threshold to better visualize individual Purkinje cells. The dendrite lengths, widths, areas, and branch numbers of each Purkinje cell (8 or 15) within a section were averaged. Measurements were then averaged across sections to obtain one representative measurement per parameter per animal. Student’s t-tests (*p* < 0.05) or a one-way ANOVA (p < 0.05) with a Tukey’s multiple comparisons post-hoc test was performed to compare the mutant and control groups at the ages studied, for ML thickness, EGL area, and the different measures of granule cell proliferation and Purkinje cell morphology. All of the histology data was plotted according to the number of animals per genotype, per age. Since each point plotted represents an animal, each point also refers to the average calculated from the pool of tissue sections.

### Locomotion

We used the CatWalk apparatus (Noldus Information Technology; Leesburg, VA) to visualize and measure the altered gait of *Car8*^*wdl*^ mice. P30 mice of both sexes were placed into the corridor of the apparatus and the placement of the paws and the body motion were recorded with a high-speed camera during locomotion (*n* = 3 of each genotype). The illuminated footprints are easily observed as the mice walk in a relatively straight line, which can be seen on the short movies provided (Additional file 1: Movie S1 and Additional file 2: Movie S2). The print area, reported as cm^2^, is a parameter that described the total floor area contacted by the paw during the stance phase. An unpaired, two-tailed Student’s t-test (*p* < 0.05) was performed to calculate the statistical significance between the footprints of the mutant and control animals. The data points plotted correspond to the average print length, width, and area per animal.

### Grip strength

We used the Ametek Chatillon (DFE/EDFE series) meter to determine the grip strength (gf/g) of *Car8*^*wdl*^ mutants (*n* = 8) compared to control (*n* = 8) mice. The forepaws of female and male adult mice (≥P60) were placed on a grated extender and then the mouse slowly pulled away from the apparatus. Measurements for grip strength were taken 3 times from each animal and then the values averaged. An unpaired, two-tailed Student’s t-test (p < 0.05) was performed to calculate the statistical significance between the grip strength of the mutant and control animals. The data points plotted correspond to the average grip strength per animal.

### Electromyography

Electromyography (EMG) was used to assess motor coordination in postnatal developing *Car8*^*wdl*^ mice and control littermates. P19–20 (*n* = 6 of each genotype) mice were deeply anesthetized with isoflurane so that 2 silver wires could be implanted into the tibialis anterior (TA) and gastrocnemius (GC) muscles of the left hindlimb. A ground wire was implanted into the neck and a head mount connecting all the wires was secured to the head with Metabond and dental cement. Mice were provided with a pre- and post-operative analgesic and given at least 2 days to recover before the recording sessions began. The mice were attached to a pre-amplifier via the EMG head mount and allowed to move freely in a glass container during the recording. Muscle activity was recorded for at least 15 min and the data was collected using Spike2 software. After selecting a 30-s interval of locomotion, as defined by the alternating firing of hindlimb muscles, TA and GC channels were rectified and a cross-correlation waveform analysis was performed in Spike2. Burst analysis was also performed on the same 30-s interval in Spike2. The probability of synchronous muscle contractions, the number of bursts over time, the number of spikes per burst, and the mean burst length in the TA muscle were averaged among all mice and compared between-groups using the Student’s t-test (*p* < 0.05). The data points plotted correspond to the average burst number, burst frequency, and burst length per TA in each mouse.

### In vivo electrophysiology in behaving mice

For head-fixed, awake recordings, P60-P90 mice were implanted with custom made headplates and a craniotomy was made above the cerebellum [52, 53]. After 72 h of recovery, mice were trained for 30 min/day in a head-fixed apparatus for 3 days before recording. Cells were recorded and categorized based on standard stereotaxic coordinates measured from Bregma [46]. Purkinje cells were identified by the presence of complex spikes. Single unit recordings were attained with 5–8 MΩ tungsten electrodes (Thomas Recording, Germany) and then the signals digitized into Spike2 (CED, England), where single units were verified with principal component analysis. Analysis of continuous traces of > 300 s was performed with Spike2, MS Excel, and MATLAB (Mathworks, Natick, MA, USA). We examined simple and complex spikes over a pre-defined period of recording. Firing pattern variability, or regularity, is defined as a measure of the consistency of time intervals between neuronal spikes (inter-spike time interval or ISI = seconds). To quantify the average variability in Purkinje cell firing pattern, the coefficient of variation of the ISI (CV) was calculated as the ratio of the standard deviation of ISIs to the mean ISI of a given Purkinje cell. We measured local regularity with the coefficient of variation of adjacent ISIs (CV2). CV2 measures firing pattern variability within a short period of two interspike intervals (CV2 = 2|ISI_n + 1_–ISI_n_|/(ISI_n + 1_ + ISI_n_)) [54]. CV and CV2 were computed and reported as mean ± standard error of the mean (SEM). Statistical analyses were performed with unpaired, two-tailed Student’s t-tests. Statistical significance is indicated in the graphs for *p* < 0.01 and *p* < 0.0001 with ** and ****, respectively. Each data point plotted represents the firing activity of a single Purkinje cell.

## Results

### Transient anatomical alterations precede motor dysfunction

**Fig. 1:**
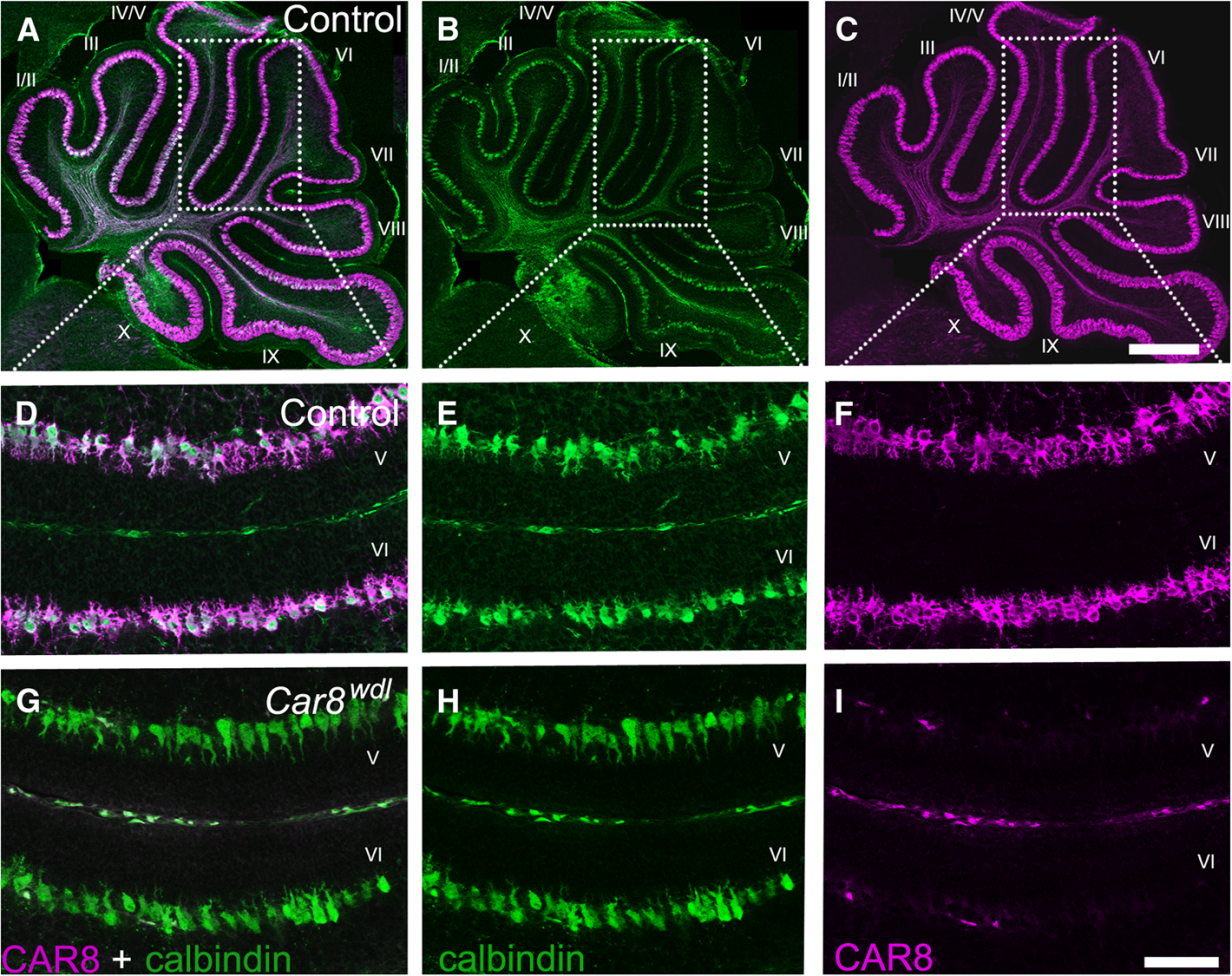
Purkinje cells in *Car8*^*wdl*^ mice do not express carbonic anhydrase VIII (CAR8). (**a**-**c**) Purkinje cells in P5 control mice (*n* = 3) express both calbindin and CAR8. (**d**-**f**) Calbindin and CAR8 are co-expressed in Purkinje cells as seen in lobules V and VI in P5 control mice (*n* = 3). (**g**-**i**) Purkinje cells in *Car8*^*wdl*^ mutant mice expressed calbindin, but not CAR8, as seen in lobules V and VI at P5 (*n* = 3). The scale bar represents 50 μm.

**Fig. 2:**
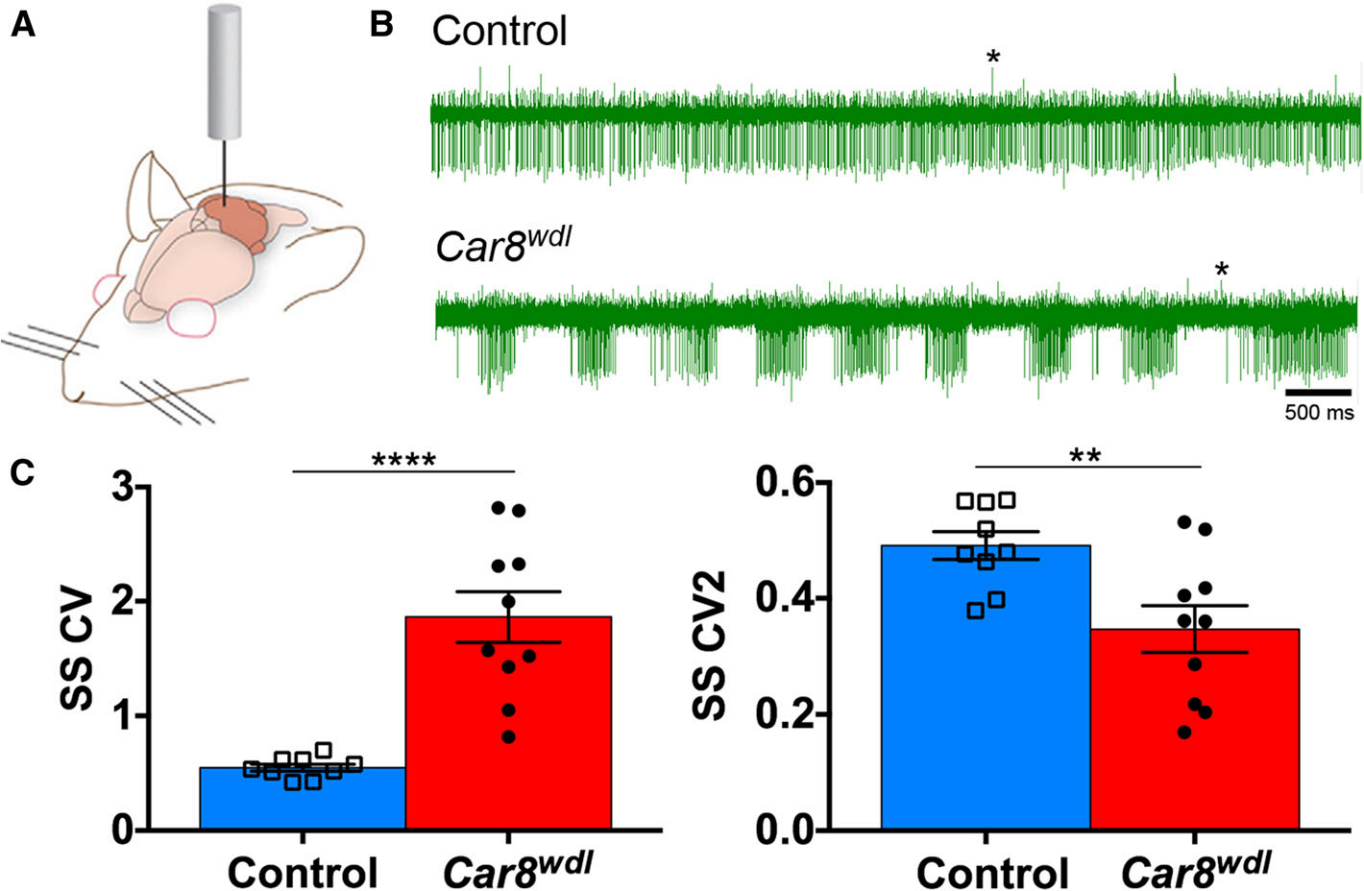
Purkinje cell firing is irregular in awake, behaving *Car8*^*wdl*^ mice. (**a**) A schematic showing a cerebellar recording in an awake mouse. (**b**) Example recordings from control (*n =* 9 cells from 3 mice) and *Car8*^*wdl*^ mutant (*n =* 10 cells from 4 mice) Purkinje cells using metal electrodes for extracellular recordings. Examples of complex spikes are labeled with asterisks. (**c**) The overall regularity and local spike-to-spike regularity are severely disrupted in *Car8*^*wdl*^ mice. These measures reflect the highly irregular activity of the mutant Purkinje cells. Abbreviations: Simple spike coefficient of variation, SS CV; Simple spike coefficient of variation of adjacent intervals, SS CV2. ** *p* < 0.01; **** *p* < 0.0001; Student’s t-test; mean ± SEM

**Fig. 3:**
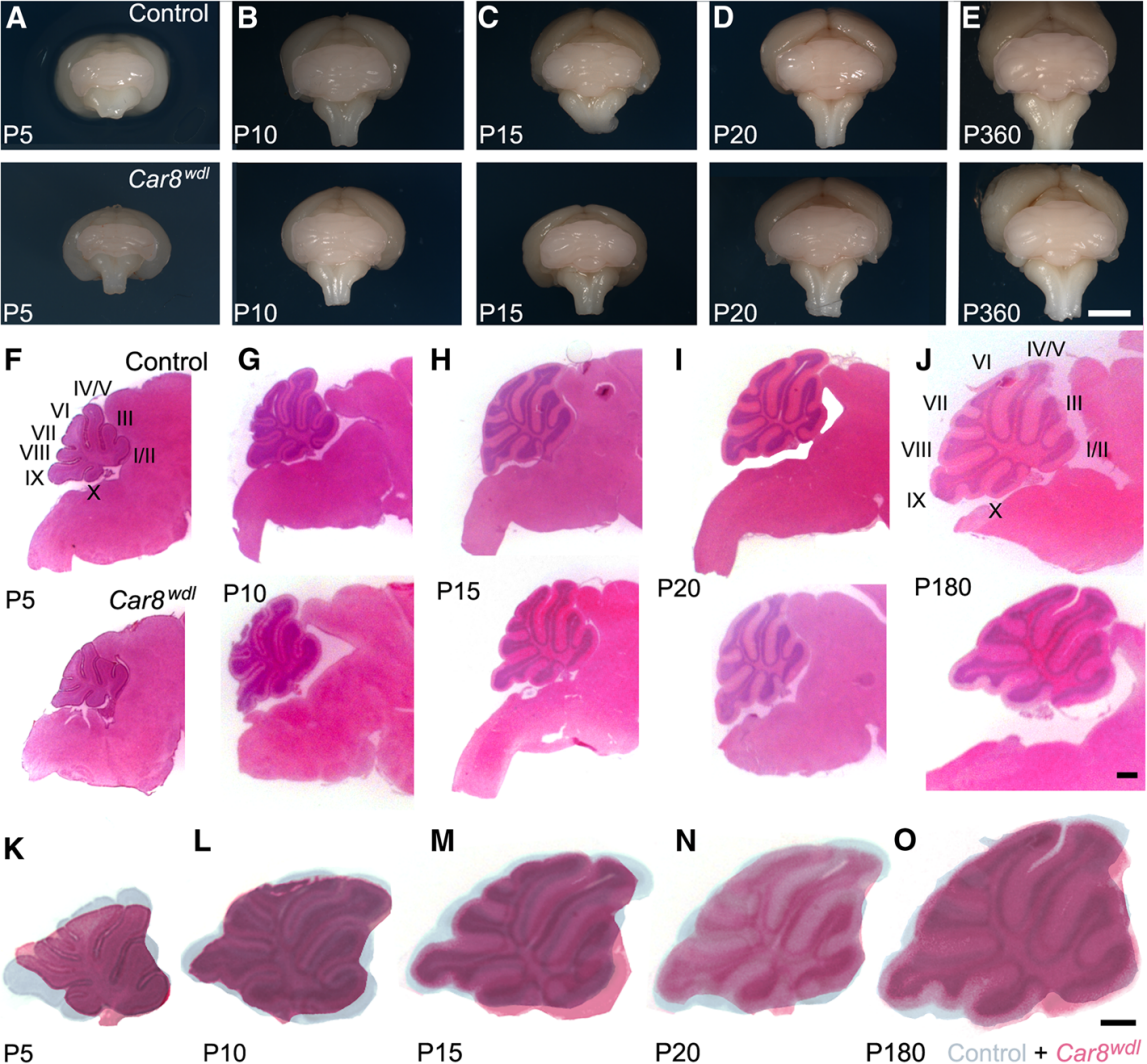
*Car8*^*wdl*^ cerebella have a normal lobule pattern but are smaller at P5. (**a**-**e**) Whole mount images of control and *Car8*^*wdl*^ cerebella at ages P5, P10, P15, P20, and adult (> 2 months) show normal lobulation in both groups. The scale bar represents 2 mm. (**f**-**j**) H&E stained tissue sections at ages P5 (*n* = 4 controls, 3 mutants), P10, P15, P20, and adult (> 2 months; P10-Adult *n* = 12 controls, 14 mutants) suggest a reduction in *Car8*^*wdl*^ cerebellar size at P5 compared to its age-matched control. The scale bar represents 1 mm. (**k**-**o**) Overlay images to compare control (blue) and *Car8*^*wdl*^ (red) cerebellar tissue sections show a transient reduction in overall cerebellar size at P5, but then is recovered by P10. The scale bar represents 1 mm

CAR8 protein is heavily expressed in Purkinje cells (Fig. 1a-f). *Car8*^*wdl*^ mice lack CAR8 in their Purkinje cells (Fig. 1g-i). As early as P14, *Car8*^*wdl*^ mice appear to walk abnormally [30]. When tested by the raised-beam task and on the accelerating rotarod at P30, *Car8*^*wdl*^ mice exhibit obvious motor dysfunction [30, 36]. Qualitative analysis of their gait using the CatWalk apparatus demonstrates their “waddles” phenotype that has features of ataxia and disequilibrium (Additional file 1: Movie S1, Additional file 2: Movie S2). These behavioral defects are supported by the presence of dysfunctional Purkinje cell activity. Extracellular in vivo electrophysiological recordings conducted in awake behaving P60 mice revealed erratic firing activity in the mutant Purkinje cells (Fig. 2; *n* = 9 cells from 3 controls, 10 cells from 4 mutants). We also previously showed that Purkinje cell firing is abnormal in anesthetized P21 *Car8*^*wdl*^ mice [30]. Erratic Purkinje cell firing with long pauses is associated with several different cerebellar disorders [2, 7, 55, 56]. The early firing defects prompted us to ask whether cerebellar structure was also affected in developing *Car8*^*wdl*^ mice. Although the morphology of the adult *Car8*^*wdl*^ cerebellum is ostensibly normal, we found that the molecular layer (ML) is significantly thinner in *Car8*^*wdl*^ at P30 [30]. We therefore decided to look even earlier than P30 to investigate whether postnatal maturation of cerebellar size and lobulation are affected in *Car8*^*wdl*^ mice. Whole mount imaging of surface morphology did not reveal obvious gross morphological differences at all of the ages analyzed (P5, P10, P15, P20, P360; Fig. 3a-e). However, a comparison of H&E stained sagittal sections revealed that *Car8*^*wdl*^ cerebella are approximately 21.5% smaller than control cerebella at P5 (Fig. 3f, k; *p* < 0.0001; *n* = 4 controls, 3 mutants). As the mice age from P10 to P180, *Car8*^*wdl*^ and control cerebella became comparable in size (Fig. 3g-j, l-o; *p* = 0.7700; *n* = 12 controls, 14 mutants). Postnatal cerebellar size was therefore transiently affected. Interestingly though, the 10 primary lobules were recognizable at all ages in *Car8*^*wdl*^ mutants, indicating that patterning is largely intact in the absence of CAR8 function. Although the reduction in size affected the whole cerebellum, regardless of lobule (Fig. 3k), we focused the remaining studies on the development of lobules V/VI for several reasons. The movement deficits in the *Car8*^*wdl*^ mutants–ataxia, dystonia, and tremor–all heavily involve limb motion, which is represented in the anterior and central cerebellar lobules that encode information for locomotion [47, 48, 51, 57–60]. More specifically, Purkinje cell firing during stepping, speed, and posture and the connectivity of lobules V/VI to the motor cortex and spinal cord via the cerebellar nuclei, validate lobules V/VI as a representative region for studying defects underlying motor disease [49, 50, 61]. Lobules VI/VI are also ideal as an experimental system because the long, straight areas of cortex allow for consistent quantification of anatomy. It is noteworthy to mention that the lobules do have different developmental timetables [62]. This also necessitated a focus on a specific subset of folds, which is helpful when following the dynamics of different cell types through several ages.

### Granule cell proliferation is impaired in *Car8*^*wdl*^ mice

**Fig. 4:**
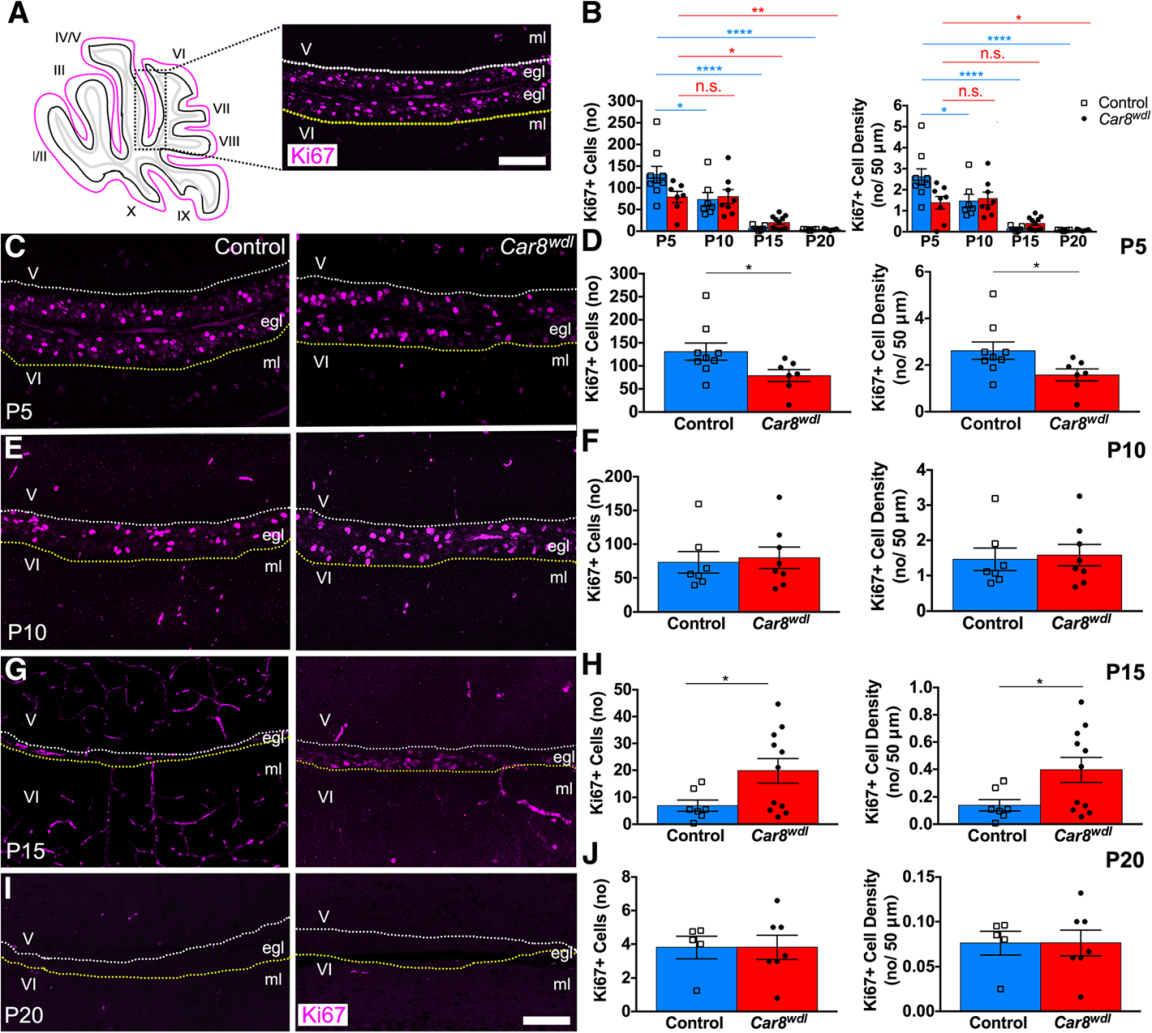
EGL proliferation in the *Car8*^*wdl*^ mutants recovers by P20. (**a**) Schematic of tissue orientation and quantification location. White and yellow dotted lines delineate inner EGL boundaries of lobules V and VI. The scale bar represents 50 μm. (**b**) Granule cell proliferation is abnormal in developing *Car8*^*wdl*^ mice. * *p* < 0.05; ** p < 0.01; **** p < 0.0001; One-way ANOVA; Tukey’s multiple comparisons post-hoc test; Mean ± SEM. (**c**-**d**) There are significantly fewer proliferating cells total and per 50 μm in the EGL of *Car8*^*wdl*^ mice (*n* = 8) than in the EGL of control mice (*n* = 9) at P5. * *p* < 0.05; Student’s t-test; Mean ± SEM. (**e**-**f**) The number (no) of proliferating cells total and per 50 μm in the EGL of *Car8*^*wdl*^ mice (*n* = 8) is not significantly different from that in the EGL of control mice (*n =* 7) at P10. *p* = 0.7691; Student’s t-test; Mean ± SEM. (**g**-**h**) There are more proliferating cells total and per 50 μm in the EGL of *Car8*^*wdl*^ mice (*n =* 11) than in the EGL of control mice (*n* = 7) at P15. * p < 0.05; Student’s t-test; Mean ± SEM. (**i**-**j**) The number (no) of proliferating cells total and per 50 μm in the EGL of *Car8*^*wdl*^ mice (*n* = 7) is not significantly different from that in the EGL of control mice (*n* = 5) at P20. *p* = 0.9930; Student’s t-test; Mean ± SEM. The scale bar represents 50 μm

During development, precisely modulating granule cell proliferation enables the cerebellum to attain the correct size. Granule cell proliferation typically peaks between P4 and P8 and is reduced from P15 to P20 due to cell differentiation and migration, which corresponds to times when cerebellar size exponentially increases and when cerebellar morphogenesis is coming to completion. (i.e. P20) [14]. *Car8*^*wdl*^ mice do not deviate largely from this timetable in that granule cell proliferation does decrease over time (Fig. 4b). Cells proliferate the most in the P5 and P10 *Car8*^*wdl*^ external granular layer (EGL) and the least towards the end of postnatal development (Fig. 4b). However, immunohistochemical analysis using Ki67 expression revealed proliferative differences between genotypes at P5 and P15. There are fewer granule cells proliferating in the EGL of P5 *Car8*^*wdl*^ mice (*p* = 0.0482) compared to the EGL of P5 control mice (Fig. 4c-d; *n* = 7 mutants, *n* = 9 controls). Similarly, the density of Ki67-positive cells is significantly reduced (*p* = 0.0482) in *Car8*^*wdl*^ EGL at P5 compared to control EGL at P5 (Fig. 4d; *n* = 7 mutants, *n* = 9 controls). Analysis of Ki67-positive cell number and density in the EGL of P10 *Car8*^*wdl*^ and control cerebella showed no significant differences (Fig. 4e-f; *p* = 0.7691; *n* = 8 mutants, *n* = 7 controls). Interestingly, we found a higher number of proliferating granule cells in the EGL of P15 *Car8*^*wdl*^ mice compared to the EGL of P15 control mice (Fig. 4g-h; *p* = 0.0467; *n* = 11 mutants, *n* = 7 controls). Despite the enhanced proliferation at P15, by P20 the number and density (*p* = 0.9930) of Ki67-positive cells in *Car8*^*wdl*^ cerebella (*n* = 7 mutants) are comparable to those in the controls (*n* = 5 controls). These data indicate that although proliferation is delayed, the temporal window of the process does not extend past the normal developmental period in which cerebellar morphogenesis is completed (Fig. 4i-j).

### Dynamic changes in the EGL and ML in postnatal developing *Car8*^*wdl*^ mice

**Fig. 5:**
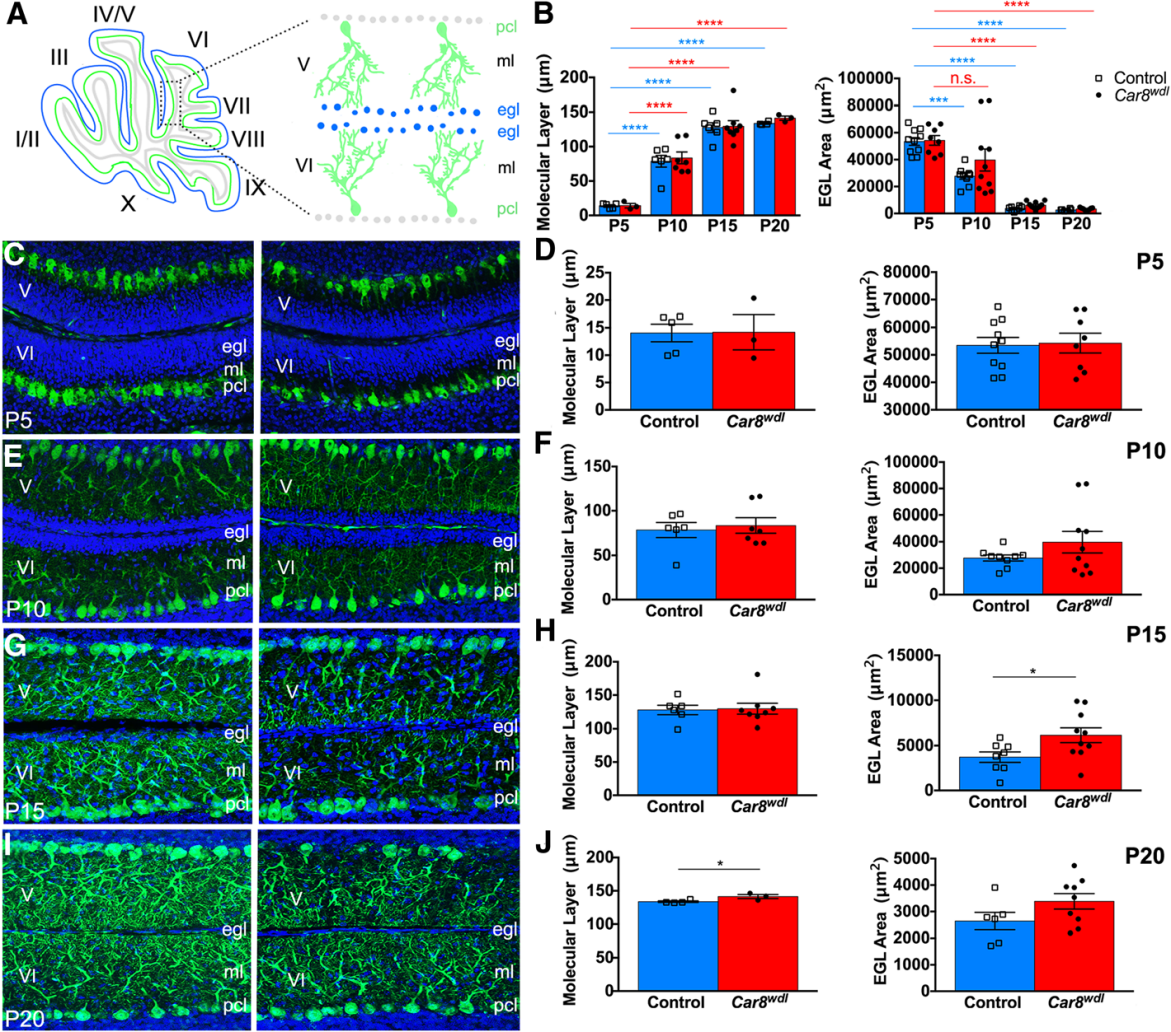
EGL size but not ML thickness recovers by P20. (**a**) Schematic of tissue orientation and quantification location. (**b**) ML thickness increases as the EGL area decreases during postnatal development in both control and *Car8*^*wdl*^ mice. *** *p* < 0.001; **** p < 0.0001; One-way ANOVA; Tukey’s multiple comparisons post-hoc test; Mean ± SEM. (**c**-**d**) Both the ML thickness (*n* = 3 mutants, *n* = 5 controls) and EGL area (*n* = 8 mutants, *n* = 10 controls) are not significantly different between *Car8*^*wdl*^ mutants and control mice at P5. ML thickness, *p* = 0.9631; EGL area, *p* = 0.8605; Student’s t-test; Mean ± SEM. (**e**-**f**) Both the ML thickness (*n* = 7 mutants, *n* = 6 controls) and EGL area (*n* = 10 mutants, *n* = 9 controls) are not significantly different between *Car8*^*wdl*^ mutants and control mice at P10. ML thickness, *p* = 0.6896; EGL area, *p* = 0.1965; Student’s t-test; Mean ± SEM. (**g**-**h**) The EGL area (*n* = 10 mutants, *n* = 8 controls), but not ML thickness (*n* = 8 mutants, *n* = 6 controls) is significantly larger in P15 *Car8*^*wdl*^ mutant mice compared to control mice. ML thickness, *p* = 0.8727; EGL area, * p < 0.05; Student’s t test; Mean ± SEM. (**i**-**j**) The EGL area in *Car8*^*wdl*^ mutant mice (*n* = 9) normalizes to controls (*n* = 6), but its ML (*n* = 3 mutants, *n* = 4 controls) is now larger at P20. ML thickness, * p < 0.05; EGL area, *p* = 0.1211; Student’s t test; Mean ± SEM. The scale bar represents 50 μm

Changes to the size of the ML or the EGL can be indicative of impaired Purkinje cell-granule cell communication. Therefore, we compared ML thicknesses and EGL areas between *Car8*^*wdl*^ mutants and control mice. Given that there were fewer cells proliferating at P5, but more cells proliferating at P15 in the mutant cerebella, we hypothesized that there would be a similar delay in the formation of the ML or the dissolution of the EGL. Compared to the ML and EGL of control mice, the ML and EGL of *Car8*^*wdl*^ mutants developed as expected (Fig. 5b). That is, the ML expanded over time to accommodate the dendritic arborization of Purkinje cells and the EGL rapidly decreased in size by P20, when granule cells are migrating rather than proliferating. Although the overall developmental trends are followed, quantification of these parameters showed fluctuations in ML thickness and EGL area at specific postnatal ages. At P5, ML thickness (*n* = 3 mutants, *n* = 5 controls) and EGL area (*n* = 8 mutants, *n* = 10 controls) are not significantly different between *Car8*^*wdl*^ cerebella compared to the control cerebella (Fig. 5c-d; *p* = 0.8605 for EGL; *p* = 0.9631 for ML). Likewise, mutant and control ML thicknesses (*n* = 7 mutants, *n* = 6 controls) and EGL areas (*n* = 10 mutants, *n* = 9 controls) at P10 are not significantly different (Fig. 5e-f; *p* = 0.1965 for EGL; *p* = 0.6896 for ML). At P15, there is still no significant difference found between mutant and control ML thicknesses (*n* = 8 mutants, *n* = 6 controls; *p* = 0.8727), but mutant EGL areas are larger (Fig. 5g-h; *p* = 0.0355; *n* = 10 mutants, *n* = 8 controls). When the timeline of EGL enlargement is compared to the timeline of granule cell proliferation, we found that this EGL enlargement precedes and overlaps with increased granule cell proliferation. Therefore, the control-sized EGL area at P5 most likely corresponds to *Car8*^*wdl*^ mice having a large, non-proliferating precursor pool. By P20, the area of the *Car8*^*wdl*^ EGL (*n* = 9) is comparable to that of P20 controls (*n* = 6; *p* = 0.1211). Yet, ML thickness is significantly increased at P20 but decreased compared to controls at P30 (P20 see Fig. 5i-j; *p* = 0.0423; *n* = 3 mutants, *n* = 4 controls; P30 see [30]). Together, the thicker EGL at P10-P15 and the fluctuating size of the ML in the *Car8*^*wdl*^ mutant mice support the hypothesis of impaired communication between cells in the developing *waddles* cerebellum. Ultimately, the data reveal that the loss of *Car8* causes transient changes in the structure of the developing cerebellar cortical layers.

### Purkinje cell dendrite morphology recovers in *Car8*^*wdl*^ mice

**Fig. 6:**
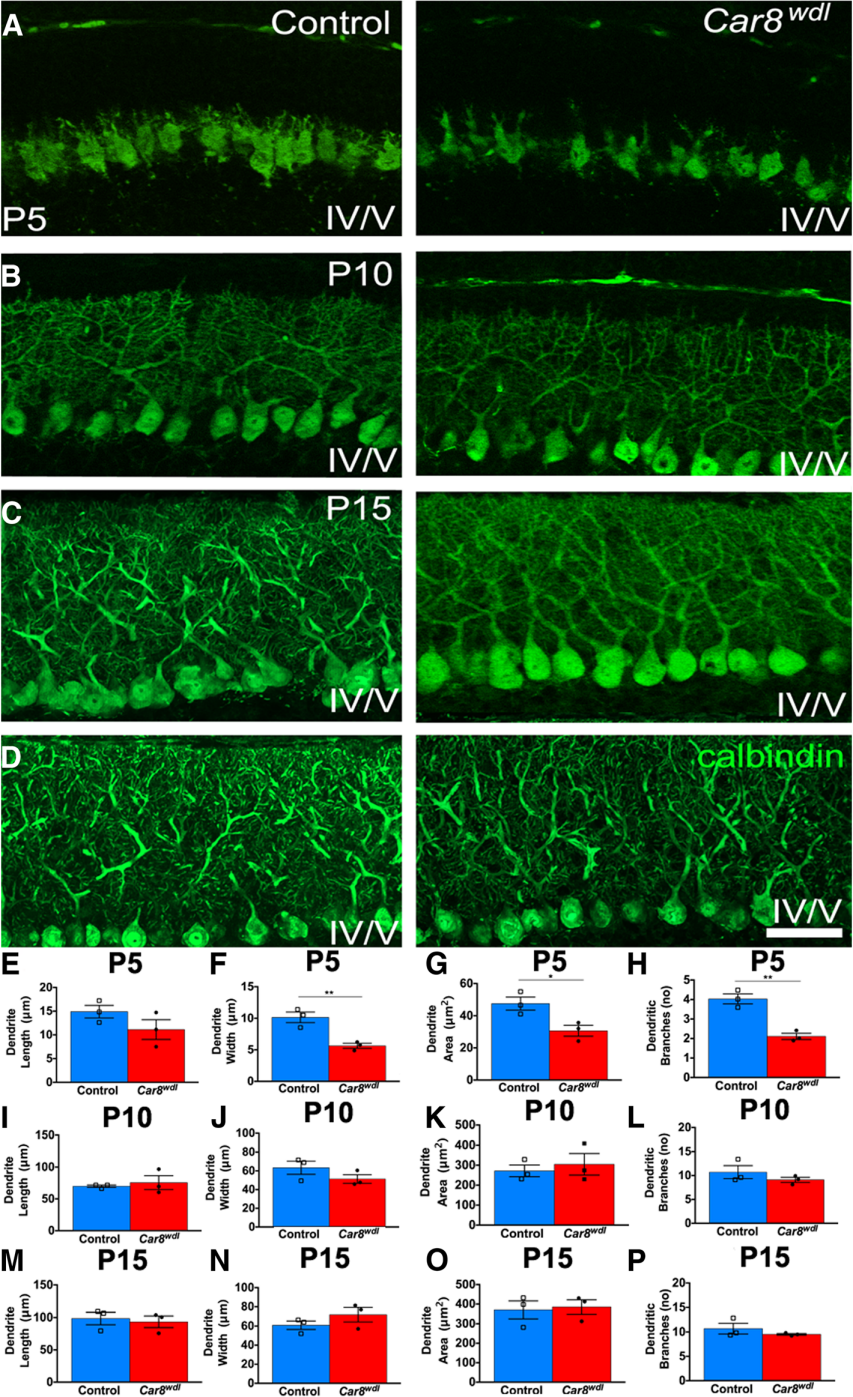
Abnormal Purkinje cell morphology at P5 recovers by P15. (**a**) Based on anatomical assessments, Purkinje cells in *Car8*^*wdl*^ cerebella are qualitatively abnormal at P5 when compared to Purkinje cells in control cerebella. (**b**-**d**) The architectural appearance of Purkinje cell dendritic arborization looks normal in *Car8*^*wdl*^ cerebella from P10 to P20. The scale bar represents 50 μm. (**e**-**h**) Quantifying dendritic tree length, width, area, and branching at P5 revealed significant differences in 3 of the 4 parameters (*n* = 3 mice per genotype). *Car8*^*wdl*^ Purkinje cells (*n* = 210) are narrower, cover a smaller area, and have fewer branches (*n* = 180 control cells) compared to controls. Dendrite length, *p* = 0.2015; Dendrite width, ** *p* < 0.01; Dendrite area, * *p* < 0.05; Branch number (no), ** p < 0.01; Student’s t test; Mean ± SEM. (**i**-**l**) *Car8*^*wdl*^ Purkinje cells (*n* = 112) no longer cover a smaller area, no longer have fewer branches, and their dendritic trees are no longer narrower at P10 (*n* = 80 control cells; *n* = 3 mice per genotype). Dendrite length, *p* = 0.6386; Dendrite width, *p* = 0.2251; Dendrite area, *p* = 0.6216; Branch number, *p* = 0.3305; Student’s t-test; Mean ± SEM. (**m**-**p**) Purkinje cell morphology (*n* = 80 mutant cells, 64 control cells) is normal across all parameters at P15 (*n* = 3 mice per genotype). Dendrite length, *p* = 0.7201; Dendrite width, *p* = 0.2766; Dendrite area, *p* = 0.8135; Branch number, *p* = 0.3486; Student’s t-test; Mean ± SEM

Although no differences in ML thickness were found at P5 between mutant and control mice, Purkinje cells qualitatively look abnormal. Calbindin staining revealed what appeared to be abnormal Purkinje cell dendritic arborization in P5 *Car8*^*wdl*^ (*n* = 3) cerebella compared to P5 control (*n* = 3) cerebella (Fig. 6a). We measured four parameters—average dendritic tree length, width, area, and average number of dendritic branches—to gain a quantitative appreciation for how robust the dendrite differences were across different ages during postnatal development. In P5 *Car8*^*wdl*^ cerebella, the width (*p* = 0.0083) and area (*p* = 0.0327) of Purkinje cell dendritic projections are significantly smaller and we found significantly fewer branches (Fig. 6e; *p* = 0.0030; *n* = 180 mutant Purkinje cells, *n* = 210 control Purkinje cells). By P10 (*n* = 3 mutants, *n* = 3 controls), Purkinje cell morphology in *Car8*^*wdl*^ mice appears normal (Fig. 6b, f; *n* = 112 mutant Purkinje cells, *n* = 80 control Purkinje cells). By P15, mutant and control Purkinje cell arborization are indistinguishable (Fig. 6c-d). Specifically, our measurements revealed that at P15 (*n* = 3 mutants, *n* = 3 controls), Purkinje cell tree length, width, area, and branch number are not significantly different between control and mutant mice (Fig. 6g; *n* = 80 mutant Purkinje cells, *n* = 64 control Purkinje cells).

### Normal mitotic progression of developing granule cells

**Fig. 7:**
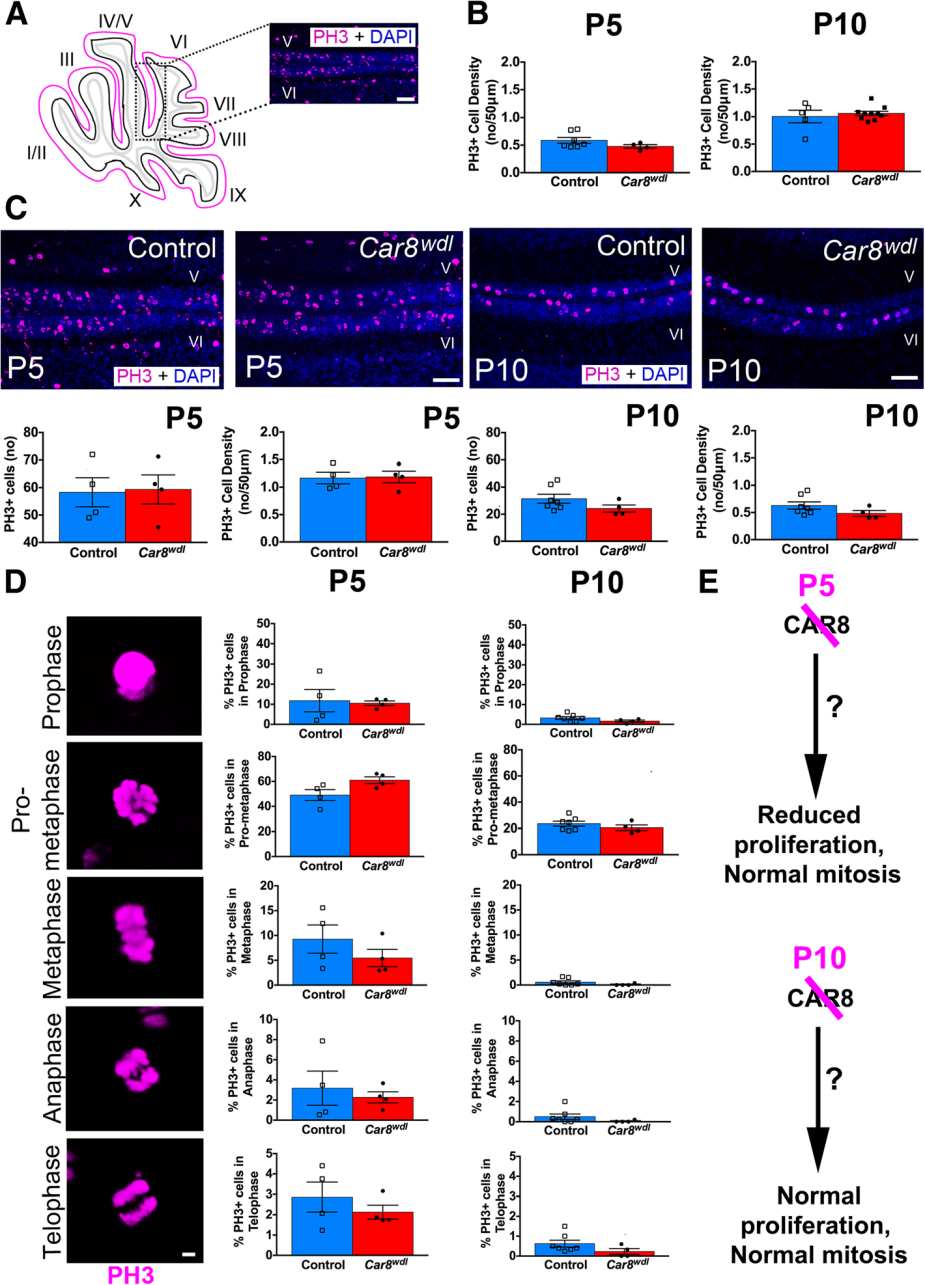
Loss of *Car8* does not delay mitotic progression in granule cell progenitors. (**a**) Schematic of tissue orientation and quantification location. (**b**) The number (no) of mitotically active cells in the P5 (*n* = 4) and P10 (*n* = 4) *Car8*^*wdl*^ EGL is comparable to that in the P5 (*n* = 4) and P10 (*n* = 7) control EGL. P5, *p* = 0.5653; P10, *p* = 0.1773; Student’s t-test; Mean ± SEM. (**c**) The number (no) and density of PH3-positive cells in lobules V-VI of *Car8*^*wdl*^ EGL (*n* = 4 at P5, 4 at P10) was not significantly different from that of lobules V-VI of control EGL (*n* = 4 at P5, 7 at P10). P5, *p* = 0.8940; P10, *p* = 0.1698; Student’s t-test; Mean ± SEM. The scale bar represents 50 μm. (**d**) *Car8*^*wdl*^ (*n* = 4) granule cells progress through mitosis normally at P5 (*n* = 4 controls) and also at P10 (*n* = 4 mutants, *n* = 7 controls). P5 Prophase, *p* = 0.8287; P5 Prometaphase, *p* = 0.0608; P5 Metaphase, *p* = 0.2968; P5 Anaphase, *p* = 0.6297; P5 Telophase, *p* = 0.3989; P10 Prophase, *p* = 0.1452; P10 Prometaphase, *p* = 0.3282; P10 Metaphase, *p* = 0.2245; P10 Anaphase, *p* = 0.2421; P10 Telophase, *p* = 0.1594; Student’s t-test; Mean ± SEM. The scale bar represents 20 μm. (**e**) Loss of CAR8 does not affect the mitotic progression of granule cells, but does transiently delay their proliferation, relative to age-matched controls

Given that Purkinje cell morphology and granule cell proliferation are transiently impaired during *Car8*^*wdl*^ development, we next sought to determine how communication between the two cell types is modified on a morphogenetic level. Due to a control-sized EGL preceding the recovery of granule cell proliferation at P10, we hypothesized that the P5 *Car8*^*wdl*^ EGL contains more cells capable of dividing. The normalization of proliferative cells at P10 could be the result of *Car8*^*wdl*^ mice having had more quiescent cells in the EGL at P5 or the result of recruiting cerebellar stem cells at P5, which have been found to occupy the PCL unless injury prompts their migration to the EGL from P0 to P5 [63, 64]. To begin to distinguish between these possibilities, we first used a Phosphohistone 3 (PH3) antibody to quantify the number of mitotically active cells as well as the percent of cells in each phase (prophase, pro-metaphase, metaphase, anaphase, telophase) at ages when the *Car8*^*wdl*^ cerebellum transitions from reduced to normalized proliferation (P5 and P10), relative to controls (Fig. 7). The PH3 antibody differs from the Ki67 antibody in that PH3 specifically labels cells in mitosis (i.e. M-phase) whereas Ki67 labels cells in interphase (G1-, S-, G2-) and in the M-phase. Neither antibody labels quiescent (G0) or differentiated cells. Although there are fewer Ki67-positive cells at P5 in lobules V/VI of *Car8*^*wdl*^ cerebella, we found that the number of PH3-positive cells in P5 *Car8*^*wdl*^ cerebella are comparable to that of P5 controls (*n* = 4 mutants, *n* = 4 controls), indicating that mitotic entry for granule cells is not delayed by the loss of *Car8* (*p* = 0.8940; Fig. 7b-c). When we calculated the percentage of PH3-positive cells in prophase (*p* = 0.8287), pro-metaphase (*p* = 0.0608), metaphase (*p* = 0.2968), anaphase (*p* = 0.6297), and telophase (*p* = 0.3989), the mutants at P5 had more cells in pro-metaphase than in telophase than controls at P5 (Fig. 7d; *n* = 4 mutants, *n* = 4 controls), albeit not by a significant amount. By P10, we found that the *Car8*^*wdl*^ mutants still have the same number of mitotically active cells in their EGLs (*p* = 0.1698), and similarly did not have delayed cell cycle progression (Fig. 7b-c, d; *p* = 0.1452% prophase, *p* = 0.3282% pro-metaphase, *p* = 0.2245% metaphase, *p* = 0.2421% anaphase, *p* = 0.1594% telophase; *n* = 4 mutants, *n* = 7 controls). Considering that there are still fewer Ki67-positive cells in P5 mutants and that communication between Purkinje cells and granule cells may be impaired, CAR8, in addition to other proteins, is likely involved in coordinating the timing of granule cell proliferation, but not through an effect on mitosis (Fig. 7e). That is, CAR8 may help to regulate granule cell progression through the G1-, S-, or G2- phases (i.e. interphase), stages of the cell cycle through which Ki67 also labels. Because the duration of the G1- and S-phases of the cell cycle are typically altered in neurons to accommodate more or less proliferation [65–67], we next investigated whether there are fewer cells in interphase in P5 *Car8*^*wdl*^ mutants.

### Sox2 is upregulated in the EGL of *Car8*^*wdl*^ cerebella

**Fig. 8:**
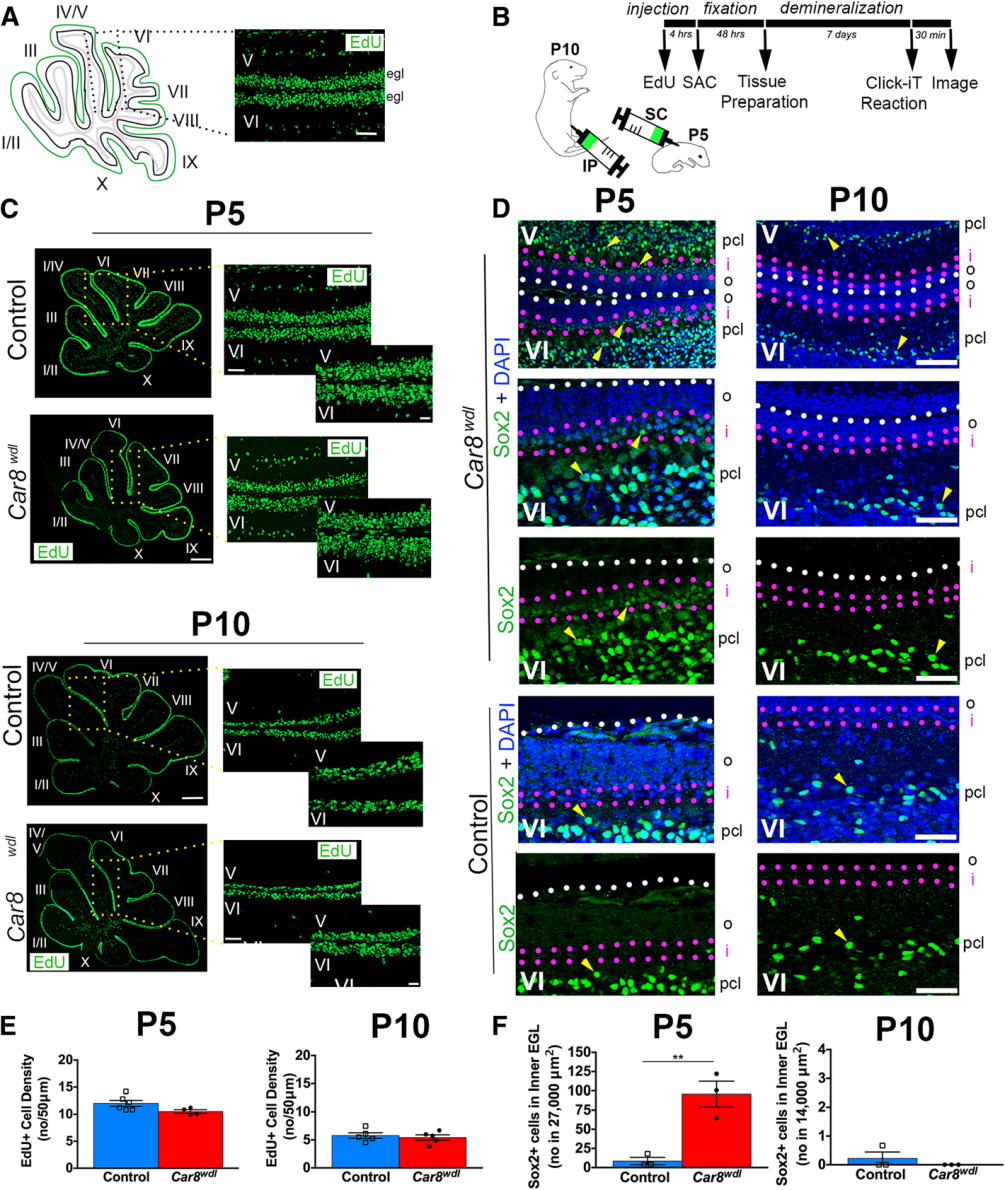
Sox2 expression is upregulated in the P5 *Car8*^*wdl*^ EGL. (A) Schematic of tissue orientation and quantification location. (B) Timeline detailing the experiment by delineating the times of the procedures performed over the course of 11 days. Two schematics are included to illustrate the method of EdU injections for P5 and P10 pups. (C,E) The number (no) of EdU-positive cells per 50 μm in lobules V/VI of *Car8*^*wdl*^ tissue is comparable to that of controls at P5 and P10. The scale bars represent 500 μm (zoomed out) and 50 μm (zoomed in). In the graphs (E), the number of cells per 50 μm that are in interphase (i.e. S-phase) are not significantly different between *Car8*^*wdl*^ and control cerebella at P5 (*n* = 6 controls, *n* = 4 mutants) or P10 (*n* = 5 controls, *n* = 5 mutants). P5, *p* = 0.0728; P10, *p* = 0.6245; Student’ t-test; Mean ± SEM. (D,F) *Car8*^*wdl*^ mice (*n* = 3) have more Sox2-positive cells (yellow arrowheads) in the inner EGL compared to control mice (*n* = 3) at P5. By P10, Sox2-positive cells are predominantly localized to the PCL and immediately adjacent areas in the *Car8*^*wdl*^ (*n* = 3) cerebellum like in the control cerebellum (*n* = 3). White dotted lines delineate the outer EGL (o). Magenta lines delineate the inner EGL (i). The scale bars represent 50 μm in (D). Quantification of the number (no) of Sox2+ cells in the P5 and P10 mutant and control inner EGL (~ 50% of the EGL, i.e. within ~ 27,000 μm^2^ or ~ 14,000 μm^2^ of the EGL) confirms the abundance of Sox2+ cells that were observed in the imaged tissue (F). P5, * *p* < 0.05; P10, *p* = 0.3739; Student’s t-test; Mean ± SEM

To determine if the occupancy of granule cells in interphase is altered at P5 but recovered by P10 in lobules V/VI, we administered EdU in vivo to P5 and P10 mutant and control pups (Fig. 8a-b). The amount of EdU that was incorporated into the EGL of P5 *Car8*^*wdl*^ mice (*n* = 4), compared to P5 controls (*n* = 6), is not significantly different at P5 (*p* = 0.1360) or at P10 (*p* = 0.7217; Fig. 8c,e). These data revealed that the EGL is correctly formed in mutants by P5 and that there are no S-phase delays in lobules V/VI. However, when taken together with data from Figs. 4 and 7, the duration of the G1- and S-phases may still be altered at P5 in *Car8*^*wdl*^ mutants because having the same number of EdU+ cells in the EGL but fewer Ki67+ cells in the EGL suggests that there are more cells in the S-phase than in G1 in P5 *Car8*^*wdl*^ mutants versus age-matched controls. At P10, the ratio of cells in G1 versus S-phase presumably normalizes, as both the number of EdU+ and Ki67+ cells in the P10 *Car8*^*wdl*^ EGL equalizes to controls. Despite the possibility of an altered cell cycle length, modifying the time spent in the G1 or S-phases would not explain why proliferation is normal at P10 or expanded at P15 in *Car8*^*wdl*^ mutants. In order for interphase to occur faster or slower, cells must already be proliferating and not quiescent. Because the P5 *Car8*^*wdl*^ EGL is equivalent in size to the P5 control EGL (Fig. 5), there is likely a pool of quiescent cells that contribute to the rescued proliferation. When we stained P5 mutant (*n* = 3) and control (*n* = 3) cerebellar tissue with Sox2, we found that there are more Sox2-positive cells in the EGL of *Car8*^*wdl*^ mice (Fig. 8d; *p* = 0.0075). The Sox2-expressing cells are localized to the inner EGL (Fig. 8d). However, by P10, the Sox2-positive cells in control and *Car8*^*wdl*^ mutant mice are densely packed within the PCL and immediately adjacent layers, consistent with the identity of these cells as future Bergmann glia and interneurons (Fig. 8d; [64, 68]). We interpret the lack of Sox2 expression in the P10 mutant EGL as an indication of earlier and completed utilization of some Sox2 cells to replenish the granule cell pool. These data suggest that either granule cell precursors ectopically express Sox2 or that Sox2-positive stem cells are recruited to the P5 *Car8*^*wdl*^ EGL to compensate for a lack of proliferating granule cells. These ideas support the hypothesis that CAR8 mediates intercellular communication and its loss triggers a rescue response for cerebellar morphogenesis to proceed in a molecular environment that is less than optimal for the normal growth and maturation of the postnatal cerebellum.

### Reorganized zonal patterning after loss of *Car8*

The anatomical defects we found in *Car8*^*wdl*^ mice transiently affect either Purkinje cell or granule cell development. Although these abnormalities disappear by the third postnatal week, it is possible that permanent defects manifest to affect behavior. For instance, several developmental processes—Purkinje cell dendritogenesis, granule cell proliferation, and granule cell migration—are interdependent. When the timing or execution of one process is altered, for example Purkinje cell dendrite outgrowth and branching, so is the timing and execution of subsequent events such as granule cell proliferation and migration, which are critical for establishing the mature cerebellar circuit [69–71].

**Fig. 9:**
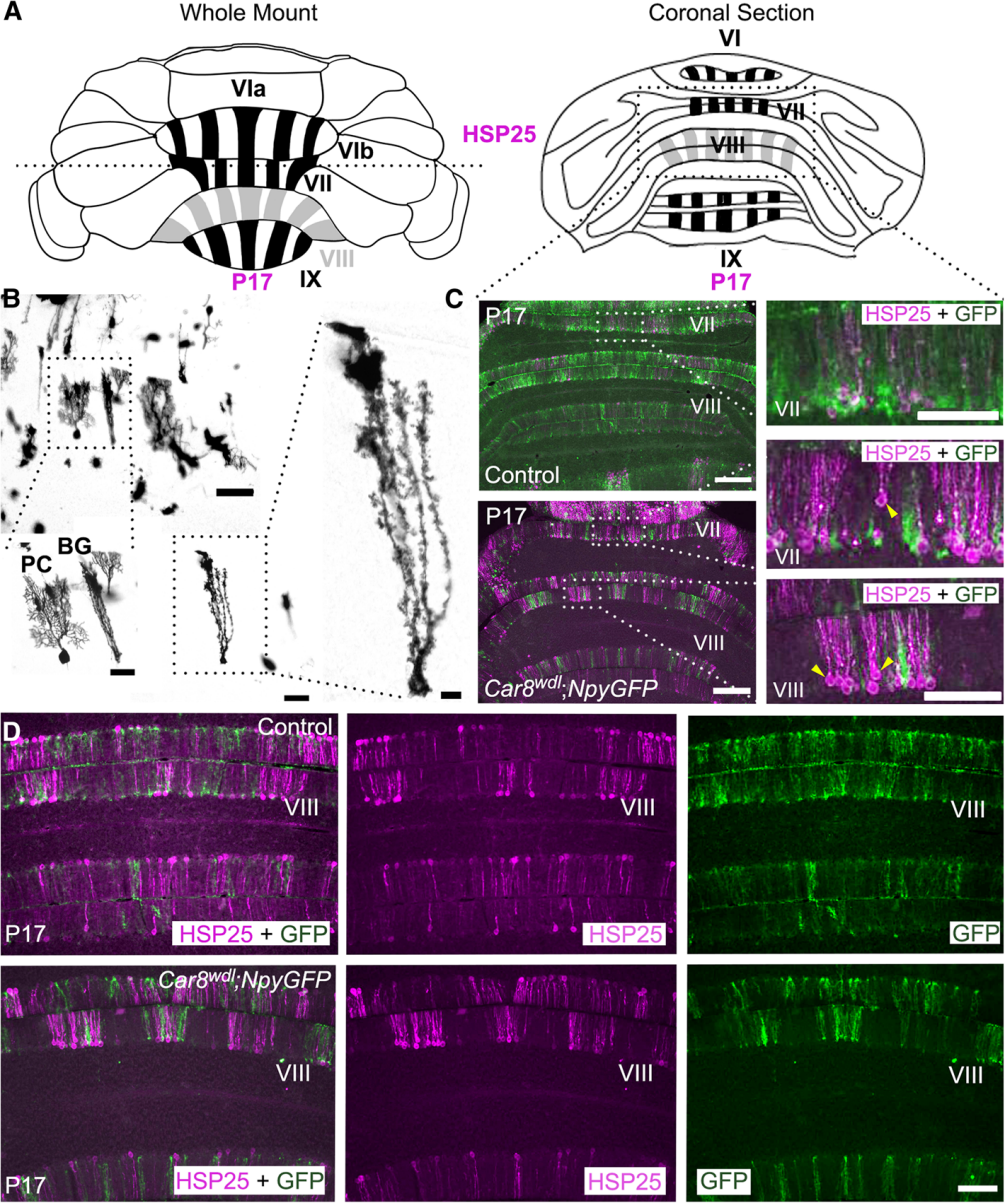
Zonal patterning of neurons and glia is delayed in developing *Car8*^*wdl*^ mice. (**a**) Schematic of HSP25 expression in a whole mount and a superficial coronally sectioned P17 posterior cerebellum. The dotted horizontal line transecting the whole mount schematic represents the location at which the cerebellum was cut to get the coronal sections below. (**b**) Modified Golgi-Cox staining in control mice showing that Purkinje cells and Bergmann glia are located together in the Purkinje cell layer (*n* = 5). Bergmann glia have long processes that facilitate granule cell migration and Purkinje cell dendritic elaboration. The scale bars represent 200 μm (Purkinje cell and Bergmann glia, top left), 100 μm (enlarged Purkinje cell and Bergmann glia (bottom left); Bergmann glia (middle)), and 50 μm (enlarged Bergmann glia, right). The Bergmann glia are labeled as BG. (**c**) Purkinje cell somas (yellow arrowheads) are abnormally aligned in *Car8*^*wdl*^ cerebella (*n* = 4), but not in control cerebella (*n* = 3) at P17. The scale bars represent 500 μm. (**d**) Double immunostaining in *Car8*^*wdl*^; *NpyGFP* transgenic mice with HSP25 and GFP reveal a zebrin II-like patterning in control (*n* = 3) and *Car8*^*wdl*^ cerebella (*n* = 4). The scale bar represents 100 μm

One process that contributes to the fine-tuning of cerebellar circuitry during development is the establishment of zones. The cerebellum is organized into a complex array of zones that are defined by lineage, gene expression, circuit connectivity, function, and behavior. Zones are experimentally examined using molecular markers and offer multiple levels of analysis, such as on the patterns, cells, and circuits of the cerebellum. In the posterior cerebellum, the small heat shock protein HSP25 labels a subset of Purkinje cells (Fig. 9a). Purkinje cells and Bergmann glia are located next to one another in the cerebellar cortex, although morphologically they are easily distinguished (Fig. 9b). At P17 in control mice, HSP25 is heavily expressed in lobules VI-IX, but only weakly expressed in lobule VIII (Fig. 9a). Using *Car8*^*wdl*^*;NpyGFP* transgenic mice (*n* = 4), we could simultaneously label Purkinje cells by immunohistochemistry and visualize the Bergmann glia with genetic reporter expression. The *NpyGFP* transgene labels a subset of Bergmann glia that share a common zonal plan to HSP25-expressing Purkinje cells in the developing and adult mouse cerebellum [72].

Here, we started by examining the tissue at the level of individual stripes and found that loss of *Car8* causes a dorsal-ventral misplacement of Purkinje cell somata within the molecular layer at P17 (Fig. 9c, yellow arrowheads). This offers another possible explanation for the somewhat paradoxical finding that although Purkinje cell dendrites are reduced in multiple parameters (Fig. 6), molecular layer thickness is increased (Fig. 5). The data suggest the possibility that abnormal stacking of Purkinje cells in the ML accounts, at least in part, for the pronounced molecular layer phenotype that we observed in the *Car8*^*wdl*^ mutants.

In many cases, alterations to zonal patterning can predict disease because of underlying impairments to the cerebellar circuit [26]. These alterations involve Purkinje cells, mossy fibers, climbing fibers, Golgi cells, unipolar brush cells, and Bergmann glia since all of these cell types and afferents use the patterning of molecular compartments to guide their own development [73, 74]. Of interest to us, was to investigate whether loss of *Car8* modifies the zonal positioning of Bergmann glia given that loss of *Car8* impacts Purkinje cell and granule cell development. In addition, loss of *Car8* has been shown to delay zone formation and alter afferent positioning [30]. Bergmann glia direct Purkinje cell dendritic outgrowth into the ML as well as granule cell migration into the inner granule cell layer (IGL) [75, 76]. Therefore, Bergmann glia are normally located next to Purkinje cells in the Purkinje cell layer and have long processes for scaffolding Purkinje cell dendrites and granule cell somata (Fig. 9b). Alterations to Bergmann glia morphology, or their positioning within the cerebellar cortex, can contribute to the pathogenesis of ataxia [77]. We predicted that the delay in HSP25 zone formation [30] might cause an equivalent delay in Bergmann glia zonal patterning. Indeed, we found that Bergmann glia zones are defective in *Car8*^*wdl*^. When comparing the distribution of HSP25-labeled Purkinje cells and GFP-labeled Bergmann glia in control and *Car8*^*wdl*^ posterior cerebella at P17, we found that there is more defined labeling in mutants, especially in lobule VIII (Fig. 9c-d). Normally, HSP25 is downregulated in lobule VIII, giving way to sharp zones only in lobules VI/VII and IX/X [78]. Despite the specific errors in Purkinje cell patterning and dorsal-ventral positioning of the somata within the tri-lamina cerebellar cortex, loss of *Car8* function does not compromise the overall compartmentalization of Purkinje cells and Bergmann glia into zones but does affect the establishment of normally robust and sharp expression of zonal marker boundaries (that is, a topographic map still forms in the *Car8*^*wdl*^ mutants, albeit defective in its fine architecture).

### Motor dysfunction develops despite rescue of cerebellar structure

**Fig. 10:**
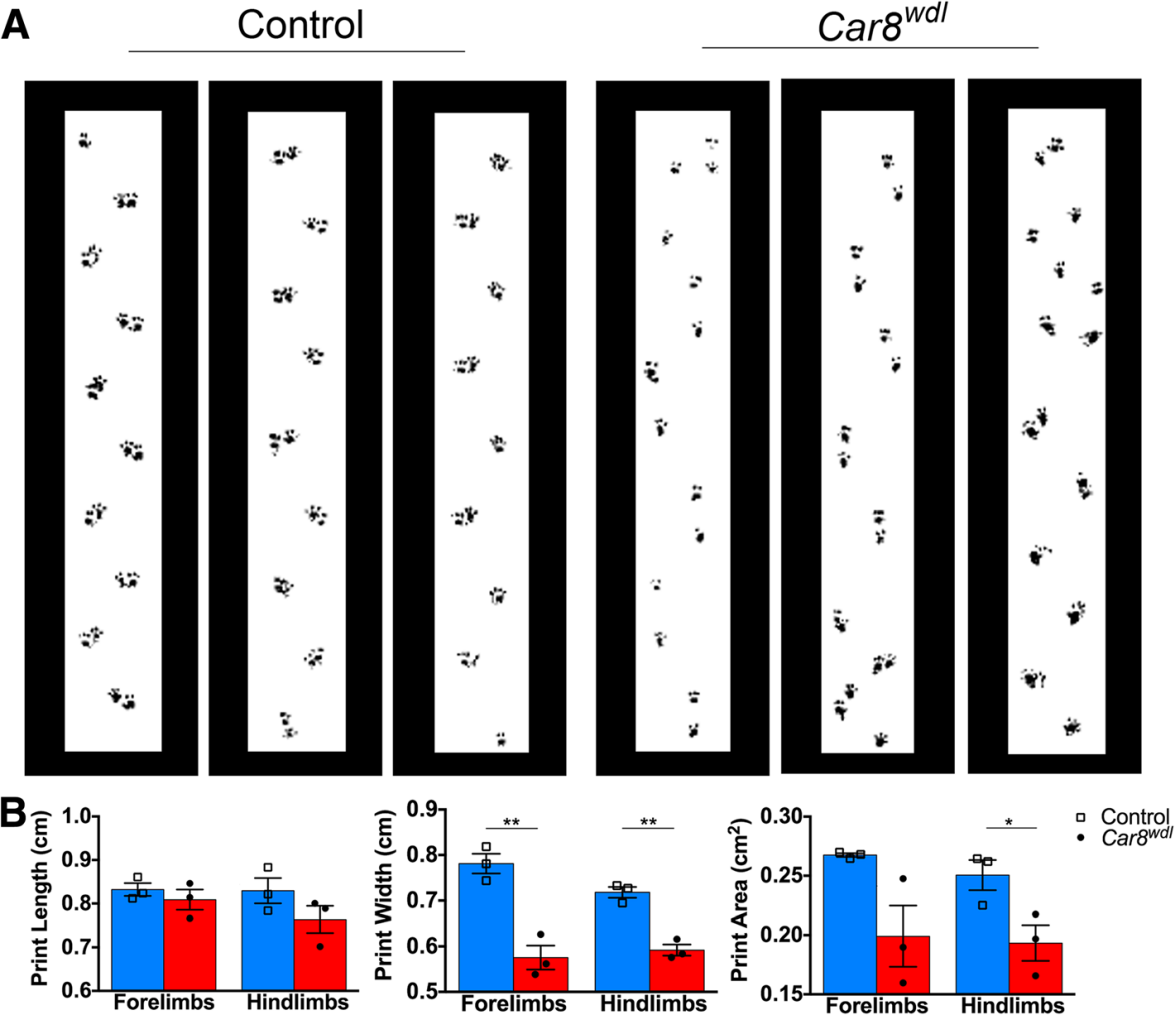
*Car8*^*wdl*^ mice have a high-stepping, tippy-toe gait that is indicative of dystonia. (**a**) Sample footprints from CatWalk for 3 control mice and 3 *Car8*^*wdl*^ mutant mice. Also see Additional file 1: Movie S1 and Additional file 2: Movie S2. (**b**) Analysis of the footprints (*n* = 3 per genotype) recorded on CatWalk show that print length is not affected in *Car8*^*wdl*^ mice (Forelimbs (*p* = 0.4444): control 0.8322 ± 0.01472 cm, mutant 0.8089 ± 0.02321 cm; Hindlimbs (*p* = 0.1977): control 0.8295 ± 0.02899 cm, mutant 0.7638 ± 0.03112 cm), but print width is reduced (Forelimbs (*p* = 0.0038): control 0.7811 ± 0.02154 cm, mutant 0.5755 ± 0.02632 cm; Hindlimbs (*p* = 0.0017): control 0.7180 ± 0.01177 cm, mutant 0.5919 ± 0.01220 cm). Accordingly, compared to control mice (Forelimbs: 0.2675 ± 0.001582 cm; Hindlimbs: 0.2505 ± 0.01276 cm), the print area is reduced in *Car8*^*wdl*^ mice (Forelimbs: 0.1990 ± 0.02576 cm, *p* = 0.0567; Hindlimbs: 0.1934 ± 0.01509 cm, *p* = 0.0445). *** p < 0.01 and **** *p* < 0.0001 Student’s t test; Mean ± SEM

**Fig. 11:**
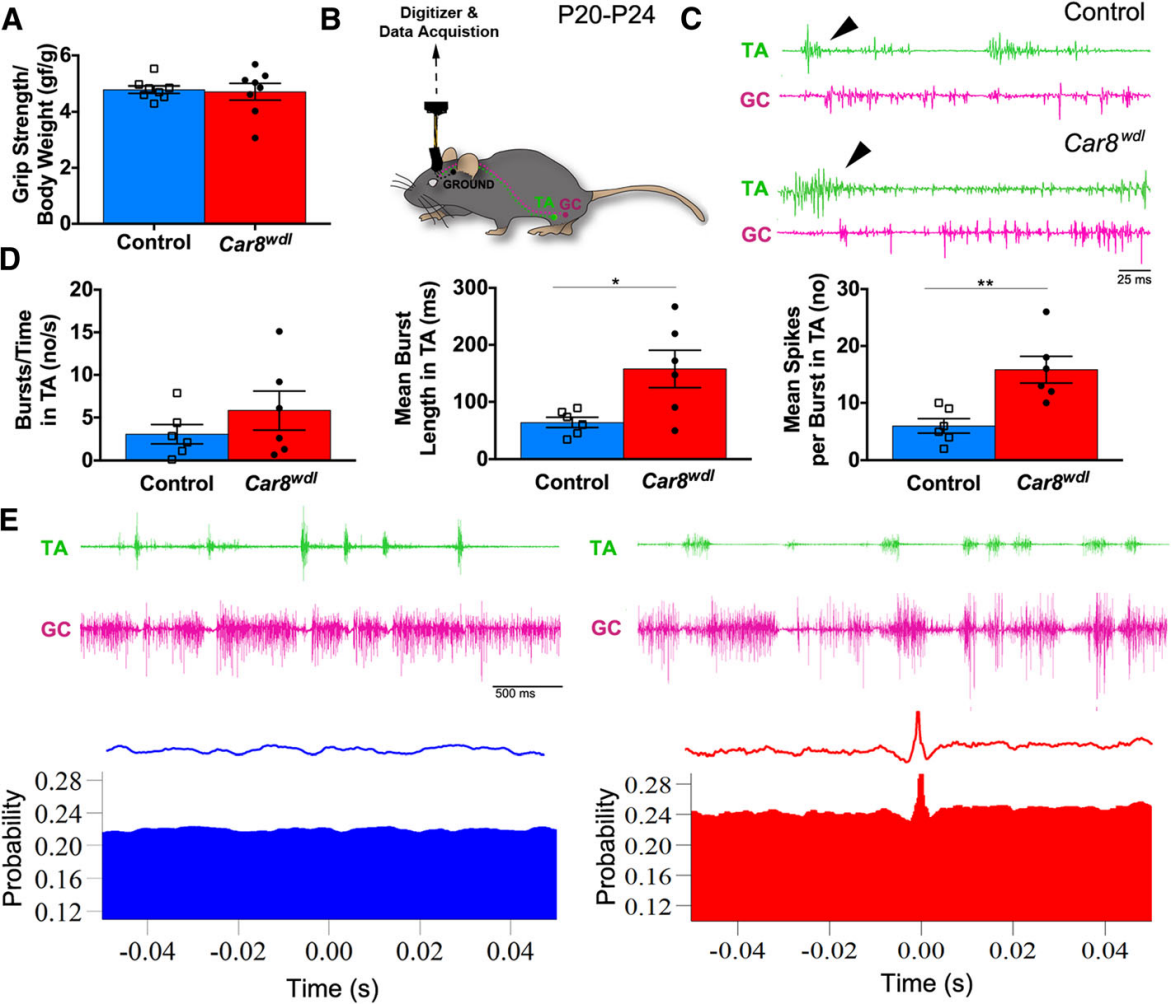
P20 *Car8*^*wdl*^ mice have normal muscle strength but abnormal muscle coordination. (**a**) Despite an ataxic gait, grip strength is not impaired in *Car8*^*wdl*^ adult mice (*n* = 8 per genotype). *p* = 0.8120; Student’s t test; Mean ± SEM. (**b**) Schematic of EMG electrode locations. TA = tibialis anterior; GC = gastrocnemius. (**c**) Bouts of TA muscle activity in *Car8*^*wdl*^ mice (*n* = 6) appear longer than in control mice (*n* = 6). The scale bar represents 25 ms. (**d**) *Car8*^*wdl*^ (*n* = 6) TA muscles fire longer individual bursts of activity, but not more frequently, than control mice (*n* = 6). The number of bursts and spikes are denoted in the graphs by the abbreviation, no. Bursts/Time, *p* = 0.3038; Burst length, * p < 0.05; Spikes/Burst, ** p < 0.01; Student’s t test; Mean ± SEM (**e**) Example EMG traces and waveform correlograms of P20-P24 control mice, showing the out of phase firing of TA and GC muscles, and of P20-P24 *Car8*^*wdl*^ mice, showing overlap between TA and GC muscle firing. The scale bar represents 500 ms

*Car8*^*wdl*^ mice are clearly ataxic in the adult [30, 36]. CatWalk analysis showed that at P30, when the cerebellar circuit has mature firing characteristics [52], *Car8*^*wdl*^ mice sway while walking (Additional file 1: Movie S1,Additional file 2: Movie S2; Fig. 10a). Analysis of the recorded footprints suggested a high-stepping, tiptoe gait in the *Car8*^*wdl*^ mice (Fig. 10b; *n* = 3 controls, 3 mutants), a behavior observed in dystonic mice [79]. Even though we observed an altered motion in two-week old mice [30], it is not clear how these defects relate to quantitative differences in activity during locomotion. The normal grip strength in adult *Car8*^*wdl*^ mutants eliminates the possibility that motor deficits are simply due to muscle weakness (Fig. 11a; *n* = 8 controls, 8 mutants). To further investigate whether motor coordination was also altered in younger mice, electromyography (EMG) recordings were performed between P20 and P24. EMG is a reliable measure of limb coordination when behavioral measures such as rotarod are impractical, which is typically the case for young mice. We used EMG recordings of the gastrocnemius (GC) and tibialis anterior (TA) muscles in *Car8*^*wdl*^ mice to acquire quantitative evidence of motor incoordination (Fig. 11b). During locomotor activity, *Car8*^*wdl*^ mice exhibited longer-lasting bouts of TA activity than control mice (Fig. 11c). Burst analysis of TA traces from *Car8*^*wdl*^ (*n* = 6) and control (*n* = 6) mice revealed that the mutants on average have significantly longer bursts (*p* = 0.0202) and more spikes per burst (*p* = 0.0040), but the same number of bursts over time (Fig. 11d; *p* = 0.3038). EMG traces from P20-P24 control mice tended to also show fewer overlapping regions between TA and GC activity compared to the EMG traces recorded from P20-P24 *Car8*^*wdl*^ mice (Fig. 11e). Cross-correlation analysis indeed showed that the P20-P24 *Car8*^*wdl*^ mice have a higher probability of muscle synchrony when the TA and GC are compared (Fig. 11d-e). On average, P20-P24 *Car8*^*wdl*^ mice (*n* = 6) have a probability of 13.29% that the TA and GC muscles would fire in synchrony compared to P20-P24 control mice (*n* = 6) that have a probability of 5.803% (Fig. 11d-e). These results suggest that agonist and antagonist muscle pairs in the hindlimbs erroneously co-contract in the *Car8*^*wdl*^ mice, and this abnormal overlap in muscle activity likely arises because of longer bursts of muscle activity. Our quantitative analysis of muscle co-contractions supports previous qualitative findings that *Car8*^*wdl*^ indeed have motor features consistent with dystonia [36].

## Discussion

Cerebellar diseases are devastating. They can cause the failure to execute a range of movements that are critical not only for fine motor skills, but also for everyday actions such as locomotion. Several of these diseases, for example ataxia, can initiate during development. Developmental cerebellar disorders involve improper circuit assembly with an ultimate consequence of disrupting function. However, in the *Car8*^*wdl*^ mouse model, circuit anatomy and patterning are initially disorganized, but surprisingly by P20, the basic architecture of the cerebellum is normal [30, 80]. Here, we report that the early postnatal defects in *Car8*^*wdl*^ mice include abnormalities in cerebellar size, Purkinje cell morphology, patterning, and granule cell proliferation. Most structural defects were transient, whereas Purkinje cell misfiring, muscle co-contractions, and motor dysfunctions persisted through adulthood.

Several morphogenetic events must occur simultaneously to enlarge the cerebellum during postnatal development. This includes Purkinje cell morphology becoming more elaborate, Bergmann glia directing Purkinje cell dendritic outgrowth into the molecular layer, and granule cells proliferating in the EGL [75, 76, 81, 82]. It is the proliferation of granule cells in the EGL that largely contributes to the rapid growth of the cerebellum [83, 84]. Since these processes are interdependent, when Purkinje cells degenerate, or Bergmann glia morphology is abnormal, granule cell proliferation and cerebellar size are affected because of compromised Purkinje cell-granule cell communication and scaffolding [82, 85–87]. Although perturbations affecting Purkinje cell, granule cell, or Bergmann glia development often have drastic effects on cerebellar size, studies have shown that both the ML and EGL are, at least in certain contexts, resilient. For instance, the size of the ML fluctuates during development due to synaptogenesis and behavioral changes [62, 88]. Through the trans-differentiation of astroglia to granule cells and the differentiation of Nestin-positive or Sox2-positive stem cells into granule cells, the EGL has the potential to recover through molecular mechanisms that reconstitute the population of cells [63, 64].

The rescue of the EGL that we found could be explained by the dynamics of GCP proliferation. Normally, GCP proliferation spans from ~E14 to ~P15 in mice [14]. During this time, GCPs have characteristic divisions. The process starts with GCPs dividing symmetrically, and then as the GCPs near differentiation, their number of divisions peak [89]. We show that decreased cerebellar size in *Car8*^*wdl*^ mice is compensated for by an atypical presence of robust granule cell proliferation at P15 (Fig. 4b,g-h). This is in contrast to control mice because normally by P15 the EGL is almost completely depleted of GCPs [15]. We also found a control-sized EGL in P5 and P10 mutants (Fig. 5b-f). These data suggest that granule cell proliferation is delayed in *Car8*^*wdl*^ mice and that a large precursor pool is likely present at P5 and available to reconstitute the EGL. This process may contribute to the homeostatic rescue of cerebellar morphogenesis in the *Car8*^*wdl*^ model. Analysis of granule cell development during mitosis and interphase indeed supports this hypothesis. We used PH3 expression, Sox2 staining, and EdU in vivo labeling to determine how loss of *Car8* transiently delays granule cell proliferation. Our findings reveal that granule cell proliferation and cerebellar size may be recovered by upregulating Sox2 expression either in granule cell precursors or through recruiting Sox2-positive stem cells to the EGL. Sox2-positive stem cells have been shown to differentiate into cerebellar granule cells in vivo and in vitro [63, 64, 90]. A Sox2-positive population of cells occupies the PCL early in postnatal development [63, 64]. This pool of multipotent progenitors decreases with age and is greatly diminished by P8 [64]. When the EGL is injured, this Sox2-postive pool is capable of repopulating granule cells by migrating from the PCL to the EGL and changing their glia and interneuron fates [64]. This migration and subsequent change in fate extends cell proliferation in the EGL into later developmental stages [64]. Therefore, one way in which Sox2-positive cells may be recruited to compensate for granule cell loss is through the modulation of signaling pathways associated with sonic hedgehog function. For example, in sonic hedgehog-dependent medulloblastoma, there are more Sox2-positive cells in the EGL, followed by more granule cells [91]. When sonic hedgehog signaling is dampened, so is the amount of ectopic Sox2-positive cells and granule cells [91]. These findings support previous data that aberrant signaling between Purkinje cells and granule cells alters morphogenesis. It is interesting to speculate that altered signaling in *Car8*^*wdl*^ mice may trigger a compensatory response from Sox2-positive cells to rescue communication between Purkinje cells and granule cells.

In addition to revitalizing the EGL during postnatal morphogenesis, Purkinje cell architecture achieves normal dimensions, and the innervation of the Purkinje cells by climbing fibers reaches typical patterns [80], but what is perhaps most interesting about the *Car8*^*wdl*^ model is that these defects are all repaired within the normal window of development. That is, cerebellar size and structure are rescued during the “critical period,” which in the cerebellum occurs until ~P17 [92]. But, Purkinje cell firing seems to lack the potential for repair in this model. There are several possibilities for how structure and function are differentially impacted in *Car8*^*wdl*^ mice. Most notably, loss of *Car8* delays granule cell proliferation (Fig. 4), and we postulate that this could lead to a delay in granule cell migration and parallel fiber formation. This delay can ultimately manifest as reduced excitatory input and ultrastructural defects in Purkinje cells, as supported by microarray studies that show a correlation between a loss of *Car8* and decreased expression of genes involved in synapse formation and maintenance [35, 93]. Previous manipulations of the EGL further support the hypothesis that transient morphological defects in developing *Car8*^*wdl*^ mice contribute to the severe phenotypes that result. For example, transiently injuring the EGL from P1-P4 to temporarily decrease granule cell proliferation, transiently prolonging the cell cycle of GCPs at P3 to delay granule cell migration, and delaying granule cell differentiation cause permanent wiring defects as well as persistent motor and vestibular deficits [69, 71, 94]. The findings that loss of *Car8* alters postnatal patterning of Purkinje cell stripes (Fig. 9) and that *Car8* modulates Purkinje cell morphology [95], further suggest the possibility that defective cerebellar firing could also arise due to intrinsic molecular defects in the mutant Purkinje cells.

Here, we show that loss of a Purkinje cell gene causes a temporary increase in ML size at P20, which could involve several circuit deformities that impact the EGL. As granule cells migrate inwards after proliferation to create the inner granule layer (IGL), they leave behind their axons, which form synapses with Purkinje cell dendrites and interneurons in an ascending order. This “stacking” process contributes to the expansion of the ML [88, 96]. In *Car8*^*wdl*^ mice, the temporary increase in ML size at P20 may be attributed to impaired synaptogenesis or perhaps to even increased ambulation. In mice, locomotion develops during the first two weeks after birth [97]. After P15, locomotion becomes more complex, with features such as the step cycle and joint biomechanics maturing to support the need for increases in the speed of locomotion and improvements in coordination [97]. In rodents, the relative amounts and type of activity as well as other behaviors such as exploration impact Purkinje cell dendritic development and synapse size [98, 99]. Therefore, increased movement from P15 to P20 in *Car8*^*wdl*^ mice, and any functional abnormalities therein, may also contribute to fluctuations in ML size.

Despite the restoration of cerebellar morphology and the main features of the circuit diagram, the function of the cerebellum is less amendable to automatic repair. Analysis of Purkinje cell firing in awake behaving mice confirms our previous report in anesthetized mice showing that *Car8*^*wdl*^ Purkinje cells fire with an irregular pattern [30], a measure of neuronal function that is currently thought to translate directly into behavioral deficits such as ataxia and dystonia because Purkinje cell excitation and spiking correlate with the stimulation of agonist/antagonist muscles during locomotor activity [6, 7, 25, 30, 59–61, 100–103]. The neuronal mechanism for how the irregular firing of Purkinje cells influences behavior likely involves their direct targets, the cerebellar nuclei. When Purkinje cells fire erratically they induce similar irregularities in the firing of cerebellar nuclei output neurons [6, 7, 79, 101]. What is intriguing about the *Car8*^*wdl*^ mice is that they exhibit ataxia (Fig. 10, Additional file 1: Movie S1 and Additional file 1: Movie S2) and tremor [30], which both presumably have origins in defective cerebellar output. Jiao et al. (2005) performed a qualitative neurological analysis and concluded that they also have appendicular dystonia [36]. In addition, Jiao et al. (2005) found that the *Car8*^*wdl*^ mice have abnormal paw placements [36]. We performed a quantitative analysis of muscle function using EMG, footprinting, and CatWalk to confirm that *Car8*^*wdl*^ mice have co-contractions of agonist and antagonist muscles in the hindlimb, abnormal stepping, and abnormal paw contacts with the floor (Figs. 10 and 11; Movie S1 and Additional file 1: Movie S2). These are all features consistent with dystonia-like behavior in rodents and using the combination of EMG, footprinting, and CatWalk allowed for a comprehensive view of movement dynamics [104, 105]. Interestingly, cerebellar function has previously been shown to impact how the hindlimb paws contact the ground. Robertson and McCollum (1991) examined the different receptive fields in the cerebellum innervated by climbing fibers [106]. The receptive fields, and by extension the climbing fibers, that are activated affect hindlimb paw placement [106]. Therefore, in *Car8*^*wdl*^ mice and in other rodent models of dystonia where climbing fiber to Purkinje cell communication is impaired [2, 7], it might be expected that erratic Purkinje cell firing accompanies abnormal stepping. Altogether, our data suggest that it is possible that the altered pattern of Purkinje cell activity in *Car8*^*wdl*^ reflects combined firing changes that culminate into motor system-wide defects that promote phenotypes such as ataxia, tremor, and dystonia.

## Conclusion

Purkinje cells play a central role during cerebellar development and they are critical for motor and non-motor functions. It is therefore not surprising that Purkinje cell defects cause diverse behavioral deficits. However, in some cases, compensatory mechanisms during development can mask the cellular and circuit deficits that are typical of diseases such as ataxia. Here, we use the *Car8*^*wdl*^ mouse model to demonstrate that even if postnatal cerebellar morphogenesis is rescued, neuronal misfiring, muscle dysfunction, and overt behavioral abnormalities can persist. Therefore, our data highlight the importance of timing during development. While the brain is equipped with many safeguards and compensatory mechanisms, short delays during critical periods may be enough to cripple subsequent circuit function and animal behavior.

## Additional files

Additional file 1: **Movie S1.** P30 Control mice exhibit normal gait. Example of a control mouse walking along the CatWalk apparatus. (M4V 324 kb)

Additional file 2: **Movie S2.** P30 *Car8*^*wdl*^ mice exhibit abnormal gait. Example of a *Car8*^*wdl*^ mouse on the CatWalk displaying disequilibrium (excessive side-to-side swaying) and ataxia (wide-based gait and lack of coordination during movement). (M4V 381 kb)

## Abbreviations

(SS) CV: (Simple spike) Coefficient of Variation
(SS) CV2: (Simple spike) Coefficient of Variation of adjacent intervals
CAR8: Carbonic anhydrase VIII
EdU: 5-ethynyl-2′-deoxyuridine
EGL: External granule layer
EMG: Electromyography
GC: Gastrocnemius muscle
H&E: Hematoxylin & eosin
IGL: Inner granule layer
IP3R1: 1,4,5-triphosphate receptor type 1
ISI: Interspike interval
ML: Molecular layer
NDS: Normal donkey serum
NGS: Normal goat serum
P: Postnatal Day
PCL: Purkinje cell layer
PFA: Paraformaldehyde
PH3: Phosphohistone 3
SEM: Standard error of the mean
TA: Tibilias anterior muscle
